# USP7 promotes chemotherapy resistance and DNA damage response through stabilizing and deubiquitinating KDM4A in bladder cancer

**DOI:** 10.1038/s41419-025-08297-2

**Published:** 2025-12-23

**Authors:** Hailang Yang, Xiaoqiang Liu, Jianqiang Nie, Shuwei Wu, Li Ma, Yi Jiang, Lizhi Zhou, Wen Deng, Qianxi Dong, Situ Xiong, Sheng Li, Fuchun Zheng, An Xie, Songhui Xu, Bin Fu

**Affiliations:** 1 Jiangxi Provincial Key Laboratory of Urinary System Diseases, Department of Urology, the First Affiliated Hospital, Jiangxi Medical College, Nanchang University, Nanchang, Jiangxi China; 2 Reproductive Medical Center, Jiangxi Provincial Maternal and Child Health Hospital, Nanchang, Jiangxi China; 3 Institute of Molecular Pathology of Nanchang University, Department of Pathology, the First Affiliated Hospital, Jiangxi Medical College, Nanchang University, Nanchang, Jiangxi China; 4 Department of Urology, Jiangxi Provincial Cancer Hospital, Nanchang, Jiangxi China

**Keywords:** Bladder cancer, Cancer therapeutic resistance, Chemotherapy

## Abstract

Bladder cancer is a common malignancy, and the insensitivity of advanced bladder cancer to cisplatin poses an imminent challenge to treatment. Our study aims to identify novel targets that mediate cisplatin responsiveness in bladder cancer. Accordingly, overexpression of the histone demethylase KDM4A in clinical cohorts was found in association with poor prognosis. Tissue culture and animal tests showed that KDM4A pis ro-proliferative in bladder cancer cells. Using co-immunoprecipitation and mass spectrometry methods, we identified that USP7 is an interacting partners in KDM4A protein complex, in which USP7 catalyzes KDM4A proteins deubiquitination that uncouples the proteasome-dependent degradation. In accordance, a positive correlation between USP7 and KDM4A protein expression was noted in bladder cancer clinical samples. Functional validation tests confirmed that USP7 and KDM4A act complementarily to drive bladder cancer cell proliferation. Importantly, cell and animal assays all evidenced that antagonizing the USP7-KDM4A axis would aggravate cisplatin-induced DNA damage and sensitize cisplatin responsiveness.

## Introduction

Bladder cancer ranks among the most prevalent malignancies of the urinary system, exhibiting a striking gender disparity with significantly higher incidence in males than females [1]. Histologically, transitional cell carcinoma represents the predominant pathological type, clinically classified as either muscle-invasive or non-muscle-invasive based on penetration beyond the muscularis propria [2–4]. Early diagnosis is critical for therapeutic outcomes [5], while treatment strategy selection remains a primary challenge for diagnosed patients. Despite diverse therapeutic approaches encompassing surgical resection, chemotherapy, and immunotherapy, the persistently high recurrence rates and platinum-based drug resistance (particularly cisplatin resistance) continue to pose major clinical challenges [2, 6–10]. Consequently, elucidating novel mechanisms to overcome cisplatin resistance has emerged as a research priority.

Recent years have witnessed growing interest in protein post-translational modification enzymes (especially demethylases and deubiquitinases) as promising therapeutic targets. Among these, lysine demethylase 4A (KDM4A) and ubiquitin-specific processing protease 7 (USP7) have been identified as key regulators in multiple cancer types [11–15]. KDM4A (also termed JMJD2A/KIA0677/JHDM3A) specifically removes methyl groups from histone H3K9me2/3, H3K36me2/3, and H1.4K26me2/3 modifications. Accumulating evidence indicates that KDM4A not only participates in fundamental cellular processes like proliferation and migration, but also critically contributes to malignant progression [15, 16]. Cutting-edge research has unveiled multifaceted regulatory mechanisms of KDM4A in solid tumors: in non-small cell lung cancer, KDM4A interacts with Stat3 to erase H3K9me3 marks and enhance FGL1 transcription, promoting metastasis [17]. In prostate cancer, SET7/9-mediated KDM4A methylation drives progression through the NPM3 signaling axis [18], while in oral squamous carcinoma, the LEF1-recruited KDM4A complex suppresses LATS2 expression to facilitate proliferation and inhibit apoptosis [19]. Notably, KDM4A also regulates genomic stability—R-2HG resulting from IDH1/2 mutations can induce telomere dysfunction through KDM4A inhibition [20]. Mechanistically, KDM4A activates the HIF1α-DDIT4-mTOR signaling cascade by reducing H3K9me3 modification at the HIF1α promoter, thereby promoting malignant phenotypes [21]. Although KDM4A overexpression in bladder cancer tissues has been documented [22], and recent findings suggest its involvement in oxidative stress regulation via SQLE transcription [23], its role in cisplatin resistance remains unexplored, warranting in-depth investigation.

As a crucial regulator of protein homeostasis, the deubiquitinase USP7 targets key proteins involved in tumor suppression, DNA repair, and immune response [24, 25]. Emerging evidence reveals USP7’s dual roles in cancer: indirectly modulating tumor suppressors like p53 through MDM2 stabilization [26], while directly promoting cancer cell proliferation and invasion, as evidenced by its overexpression correlation with aggressive phenotypes in oral squamous cell carcinoma [27] and hepatocellular carcinoma [28]. Particularly noteworthy is USP7’s association with chemotherapy resistance: in nasopharyngeal carcinoma, USP7 mediates cisplatin resistance through the KDM5B-ZBTB16/TOP2A axis [29]; whereas in paclitaxel resistance, it regulates PLK1 protein degradation [30]. However, systematic investigations of USP7’s functional mechanisms in bladder cancer remain lacking, and deeper understanding could provide novel therapeutic insights.

This study focuses on deciphering molecular mechanisms to ameliorate cisplatin resistance in bladder cancer. Through comprehensive analysis of KDM4A and USP7 expression patterns and functional interplay in clinical specimens, we systematically investigated their roles in tumor proliferation and cisplatin resistance. Our key findings demonstrate that USP7 small-molecule inhibitors not only downregulate KDM4A expression but also significantly potentiate cisplatin’s therapeutic efficacy. These discoveries provide valuable experimental evidence and potential therapeutic targets for developing novel chemosensitizing agents in bladder cancer treatment.

## Materials and methods

### Cell lines and cell culture

293 T cell line and the T24 bladder cancer cell line were cultured in DMEM medium, while the 5637 bladder cancer cell line was cultured in RPMI-1640 medium. All culture media included 10% FBS, 100 U/mL of penicillin, and 0.1 mg/mL of streptomycin. All cell lines were routinely tested for mycoplasma contamination using PCR-based assay.

### Histopathology immunohistochemical (IHC) staining

Briefly, bladder cancer tissues were fixed in 10% neutral buffered formalin for overnight, and then processed and embedded in paraffin using standard procedures. Paraffin-embedded tissues were sectioned (5 µm), deparaffinised, repaired with citrate antigen repair solution, stained with (KDM4A, USP7, Ki-67 and r-H2AX) antibodies, and finally stained with hematoxylin for nuclei at the Clinical Medical Research Centre of the First Affiliated Hospital of Nanchang University. Histological analysis of stained tissue was performed independently by two pathologists. IHC analysis of KDM4A and USP7 was scored on the basis of staining intensity (scale of 0–3) and percentage of positive cells (scale of 1–4). The total score (ranging 0–12) was obtained with the following calculation formula: staining intensity * percentage of positive cells. Collection of IHC tissue samples at the First Affiliated Hospital of Nanchang University was subjected to internal review and approval by the Ethics Committee (Registration Number: (2022)CDYFYYLK (11–031).

### Immunofluorescence (IF) staining

Cells were seeded at a density of 1 × 10^4^ cells per well in 24-well plates. After recovery, cells were rinsed twice with PBS, fixed with 4% paraformaldehyde, and washed with PBST. Permeabilization was performed with 0.5% Triton X-100 for 20 min, followed by washing with PBST and blocking with 5% BSA for 2 h. Primary antibodies were added for overnight incubation at 4 °C. After washing with PBST, secondary antibodies were added for 2 h of dark incubation at room temperature. DAPI staining was done with 5 min of dark incubation at room temperature, followed by washing with PBST. Ultimately, images were captured with fluorescence microscopy and confocal microscopy.

### Western blotting

After lysis of cells or tissues for protein extraction, total proteins were subjected to quantification and normalization, followed by preparation with sample loading buffer and boiling. Prepared samples were resolved on SDS-PAGE gels for electrophoretic separation. Next, proteins were transferred to PVDF membranes and blocked with 5% BSA for 1 h. Primary antibody incubation was conducted for overnight at 4 °C. On the following day, the blots were washed with TBST, incubated with secondary antibodies for 1 h at room temperature, and then washed again in TBST. Eventually, development was performed with the ECL chemiluminescent reagents to obtain images.

### GST-pull down assay

The GST-USP7 expression plasmid was transformed into BL21 competent cells and incubated at 37 °C for 1 h. Next, the transformants were spread onto LB agar plate containing ampicillin for overnight incubation. Single colonies were picked for liquid incubation in LB medium and the culture were carried out at 37 °C on a shaker until OD600 reaches 0.6–8. Protein expression was induced by adding IPTG at a final concentration of 0.1 mM for overnight incubation at 16 °C. Next, the cells were harvested for lysis and the lysates were incubated with GST magnetic beads at 4 °C for overnight to capture the target proteins. The beads were then washed to remove non-specific protein and the GST-USP7 proteins were released by incubation with GSH elution buffer for 30 min. Finally, the supernatant was collected and protein content was evaluated by SDS-PAGE.

### Subcutaneous tumor model in BALB/c nude mice

BALB/c nude female mice (4–6 weeks old) were purchased from Charles River Laboratories (Beijing, China). All animals used in this study were humanely managed in accordance with applicable regulations, policies, and guidelines related to animals. Experimental procedures using animals were all approved by the Institutional Animal Care and Use Committee of the First Affiliated Hospital of Nanchang University (registration number: CDYFY-IACUC-202305QR020). According to the requirements of the Institutional Animal Care and Use Committee of the First Affiliated Hospital of Nanchang University, the maximal tumor size should not be larger than 1.5 cm^3^. And in this study, the maximal tumor size did not exceed the maximum requirement. For each indicated group of T24 tests, 1 × 10^6^ cells were mixed with Matrigel (1:1) for subcutaneous injection into the flanks of mice. Tumors were measured every 7 days using calipers and tumor volume was calculated with the following formula: length × width × width × 0.5. Tumor tissue was subjected to paraffin embedment and data were analyzed with a two-tailed Student’s t-test.

### Statistical methods

Statistical analyses were performed with the SPSS software and graphical representations were generated with the R software version 4.1.1. For comparison between test groups, we employed t-tests, one-way ANOVA, or two-way ANOVA, accordingly. Results were considered statistically significant if the computed p-value was less than 0.05.

## Results

### KDM4A is over-expressed in bladder cancer tissues and its over-expression is associated with poor prognosis

**Fig. 1:**
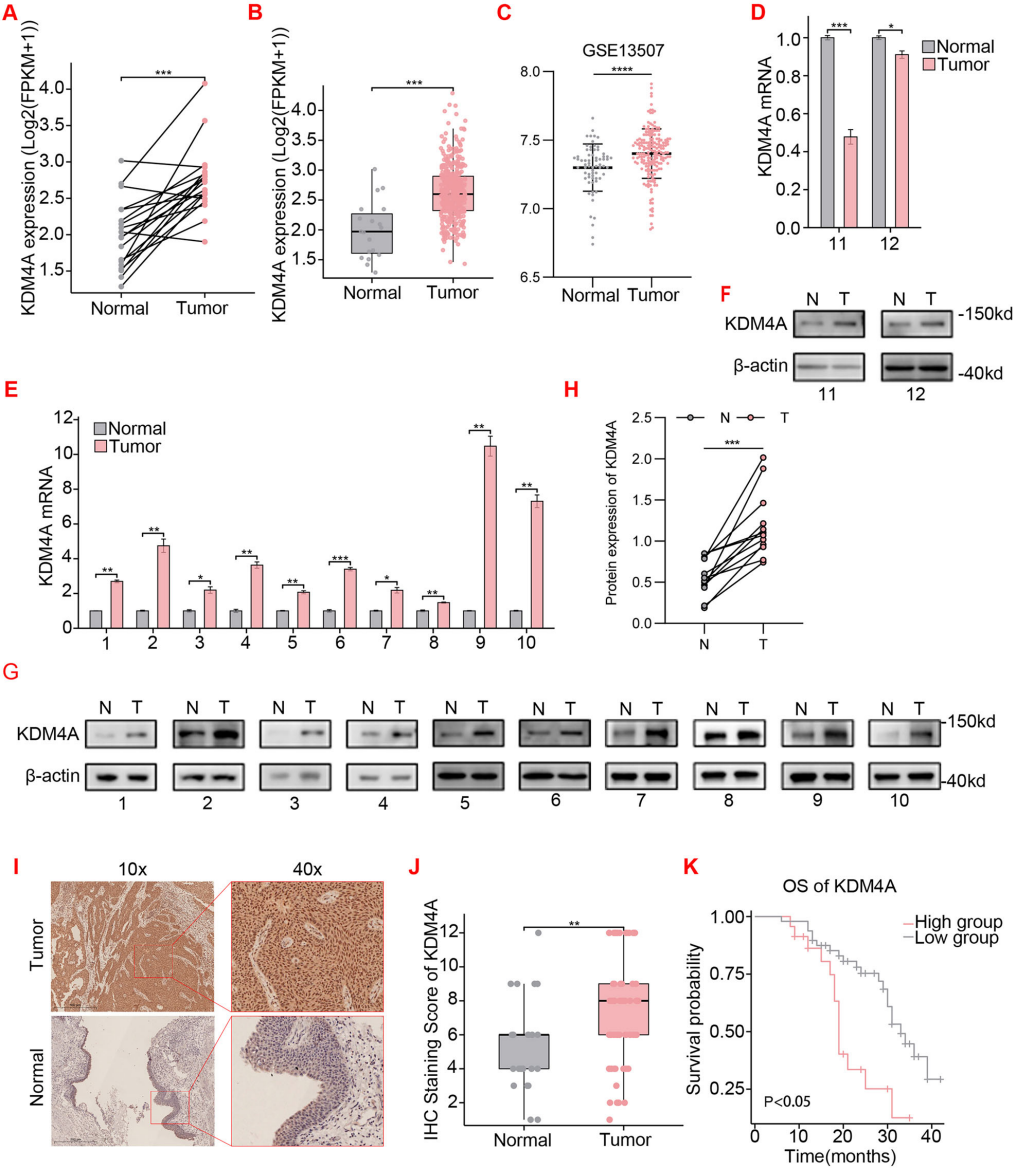
KDM4A is over-expressed in bladder cancer tissues and its over-expression is associated with poor prognosis. **A** KDM4A expression in bladder cancer and paired para-cancerous (normal) tissues from TCGA-BLCA dataset (https://portal.gdc.cancer.gov, FPKM format of TCGA-BLCA, n = 19, *p* < 0.001). **B** KDM4A mRNA expression in TCGA-BLCA dataset on unpaired bladder cancer(n = 412) versus normal adjacent tissues (NATs) (n = 19) (FPKM format of TCGA-BLCA*, p* < 0.001). **C** KDM4A expression in bladder cancer and paired para-cancerous (normal) tissues from GSE13507 dataset. **D–H** RT-qPCR analysis of KDM4A mRNA and Western blotting (WB) assay of KDM4A proteins in 12 pairs of bladder tumors (T) versus NATs (N). In both assays, β-actin was used as internal control. **I** Representative immunohistochemical (IHC) staining images of KDM4A proteins in bladder cancer versus normal bladder tissues (10x lens and 40x lens). **J** Statistics analysis on IHC staining of KDM4A proteins in bladder tumors (n = 71) versus NATs (n = 23, *p* < 0.01). **K** Prognostic Kaplan-Meier survival analysis curves of patients with high and low KDM4A expression (Log-rank, HR = 0.361, *p* = 0.001). OS: overall survival. (* P < 0.05, **P < 0.01, *** P < 0.001, **** P < 0.0001*, ns=no sign)*.

Recent studies have demonstrated the over-expression of multiple histone demethylases in various cancers, including breast cancer, prostate cancer, and lymphoma [31, 32]. We here first analyzed bladder tumors in TCGA clinical datasets (https://portal.gdc.cancer.gov) and identified over-expression of KDM4A transcripts in bladder cancer tissues as in comparison to normal adjacent tissues (NATs) (Fig. 1A, B). In addition, we also found that KDM4A expression is increased in bladder cancer tissues by analyzing the GSE13507 dataset (https://www.ncbi.nlm.nih.gov/geo/)(Fig. 1C).

To visualize KDM4A protein expression in clinical samples, we collected 12 pairs of surgically resected fresh bladder cancer tissues versus normal bladder tissues as control. To gain insight into the alignment between expression of KDM4A transcripts and proteins, we next performed RT-qPCR and found that KDM4A mRNA was over-expressed in 10 (#1 - #10) out of 12 pairs of cancerous tissue (Fig. 1E). In accordance, Western blotting (WB) assay based on normalized tissue proteins revealed over-expression of KDM4A proteins in these 10 bladder cancer tissue samples (Fig. 1G). Importantly, the remaining two clinical sample pairs (#11 and #12) obtained decrease in KDM4A transcripts but increase in KDM4A proteins (Fig. 1D–F). Quantitative analysis of KDM4A expression immunoblot images revealed that KDM4A protein expression was elevated in bladder cancer tissues (Fig. 1H). IHC showed over-expression of KDM4A proteins in the cancerous tissues (Fig. 1I, J). Next, we divided the patients into high-expression and low-expression groups according to the median KDM4A expression level detected by IHC and performed Kaplan-Meier survival analysis. As shown, bladder cancer patients with higher KDM4A expression had a worse prognosis (Fig. 1K). However, based on clinical baseline analysis of the TCGA-BLCA dataset, KDM4A mRNA expression was not associated with T stage, N stage, M stage, or pathological grade (Supplementary Table 1). Furthermore, clinical analysis of 71 cases using IHC staining was consistent with the TCGA-BLCA data (Supplementary Table 2).

### Knockdown of KDM4A inhibits the proliferation of bladder cancer cells and cisplatin resistance

**Fig. 2:**
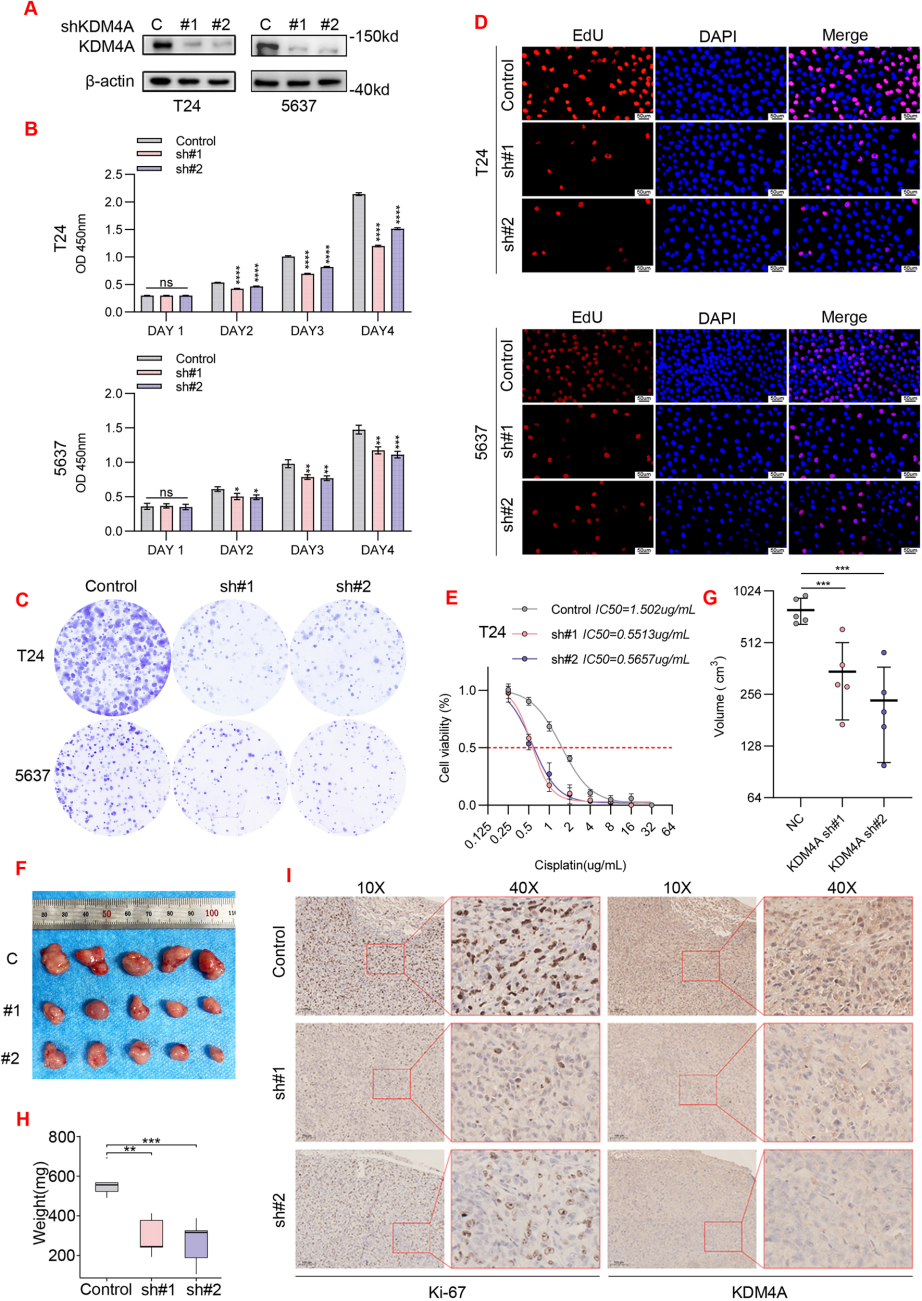
Knockdown (KD) of KDM4A inhibits the proliferation of bladder cancer cells. **A** T24 and 5637 stable cells were generated by infection of lentiviral vectors expressing control versus two KDM4A-specific shRNAs (C, #1 and #2). Total proteins were normalized for WB assay. Established T24 and 5637 stable lines were subjected to monitor in vitro KDM4A-KD effects on cell proliferation with CCK8 assay (**B**), clonal formation assay (**C**), and EdU assay (**D**). **E** IC50 of T24 cells for 48 h in cisplatin. Mice xenografts were generated with established T24 stable lines to examine the in vivo KDM4A-KD effects on tumor growth (**F**), tumor volume (*** *p* < 0.001) (**G**), tumor weight (**H**), and IHC staining (**I**). (* P < 0.05, **P < 0.01, *** P < 0.001, **** P < 0.0001, *ns=no sign)*.

The above observations linked KDM4A-OE with unfavorable prognosis, granting its candidacy as an oncogene in bladder cancer. To investigate the biological functions of KDM4A on cell proliferation in bladder cancer, two independent KDM4A-specific short hairpin RNAs (sh#1, sh#2) were introduced into T24 and 5637 cell lines via lentivirus infection. WB assay based on normalized total proteins indicated effective knockdown (KD) of KDM4A expression (Fig. 2A). Next, we took a series of cellular tests to demonstrate that KDM4A-KD effectively attenuated T24 and 5637 proliferation in CCK8 assay (Fig. 2B), plate cloning assay (Fig. 2C and Supplementary Fig. S1A) and EdU assay (Fig. 2D and Supplementary Fig. S1B). Knockdown of KDM4A leads to decreased resistance of bladder cancer cells to cisplatin (Fig. 2E and Supplementary Fig. S1C). Overexpression of KDM4A promotes bladder cancer cell proliferation and cisplatin resistance (Supplementary Fig. S1E–G).

Consistently, in vivo studies in the mouse T24 xenograft model evidenced that KDM4A-KD led to a significant decrease in tumor growth rate and tumor burden (Fig. 2F, G, H). Additionally, IHC staining of Ki67 further validated that KDM4A-KD indeed mitigated the proliferative population (Fig.2I). Collectively, these findings ratified that KDM4A functions as a bladder cancer driver in both in vitro and in vivo settings.

### Identification of USP7 as a binding partner in KDM4A protein complex

**Fig. 3:**
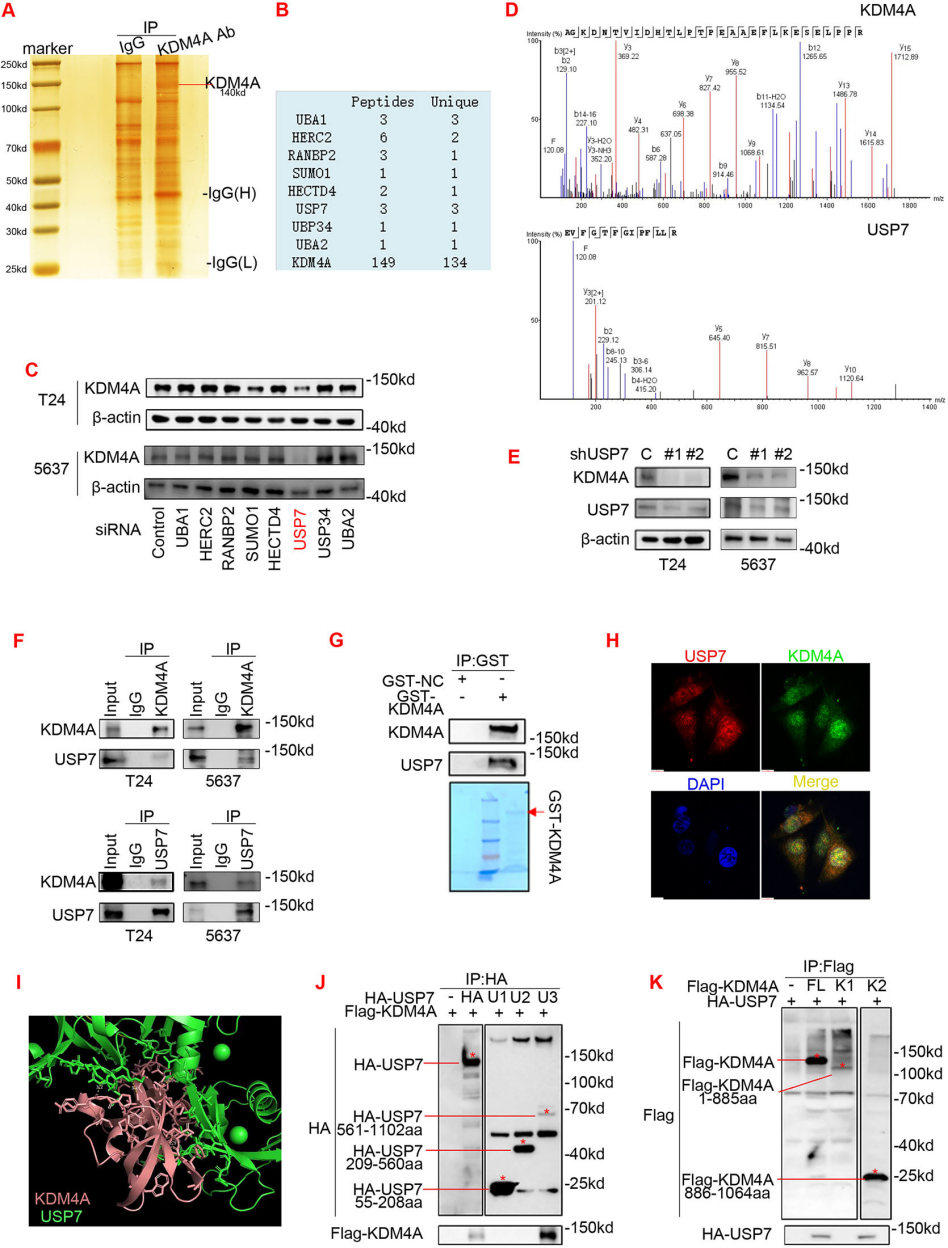
Identification of USP7 as a binding partner in KDM4A protein complex that sustains KDM4A protein expression. **A** 293 T cells were subjected to co-immunoprecipitation (co-IP) with Flag antibody. The precipitated products were resolved on SDS-PAGE for silver staining. **B** List of major hits in mass spectrometric (Mass-Spec) analysis. **C** T24 and 5637 cells were transfected with siRNA for indicated 8 genes and total proteins were normalized for WB assay of KDM4A expression. **D** Unique peptides for KDM4A (upper panel) and USP7 (lower panel) identified in Mass-Spec analysis. **E** T24 and 5637 stable lines were generated upon lentiviral infection of control versus USP7-specific shRNA (C, sh#1, and sh#2). Total proteins were normalized for WB assay of USP7 and KDM4A expression. **F** T24 and 5637 cell lysates were subjected to co-IP assay IgG (control) versus KDM4A or USP7 antibody. WB assay was performed to resolve the precipitated products and input control. **G** Purified recombinant GST-KMD4A proteins were subjected to precipitation with lysates of HEK293T cells that were transfected with HA-USP7. Co-IP products were subjected to WB assay with GST-tag versus HA-tag antibodies. Bottom, recombinant GST-KDM4A proteins were isolated from bacteria culture and resolved by SDS-PAGE and Coomassie blue staining. GST-NC: negative control with empty vector. **H** T24 cells were subjected to immunofluorescence (IF) assay with confocal microscopy, to locate intracellular distribution of KDM4A (green) and USP7 (red); nuclei were stained with DAPI (blue). **I** Graph presentation of molecular docking model to display the calculated binding interface between KDM4A and USP7. **J**, **K** HEK293T cells were co-transfected with indicated HA-USP7 versus and Flag-KDM4A constructs and then subjected to co-IP and WB assays.

The above findings supported overall over-expression of both KDM4A transcripts and proteins in bladder cancer. However, in two cases (#11 and #12), there was deviation between expression of KDM4A proteins and transcripts, suggesting the involvement of additional key factors. To systemically identify the critical regulators of KDM4A proteins, we sought affinity approaches that would expose interacting partners in protein complex. Accordingly, we over-expressed Flag-KDM4A construct in 293 T cells for co-immunoprecipitation (co-IP) test that contains highly stringent wash steps in the protocol. The co-IP products were next resolved by SDS-PAGE and differential protein bands were identified through silver staining (Fig. 3A). Subsequently, excised bands were subjected to mass spectrometry (mass-spec) analysis, exposing eight candidate regulators of KDM4A proteins (Fig. 3B). Upon interfering with these genes with specific siRNAs in T24 and 5637 cell lines (Supplementary Fig. S1H), WB assay was conducted to demonstrate that USP7-KD specifically reduced KDM4A proteins (Fig. 3C). Consistently, USP7 was also found in the peak map identified by mass spectrometry (Fig. 3D).

To solidify these findings, we infected T24 and 5637 cells with two independent USP7 shRNA (sh#1 and sh#2) to verify that USP7-KD indeed down-regulated KDM4A protein expression in bladder cancer (Fig. 3E). Next, we conducted co-IP experiments to validate that USP7-KDM4A protein interaction occurred in T24 and 5637 cells (Fig. 3F). Moreover, GST-pull down assay further confirmed the direct binding between USP7 and KDM4A proteins happened in vitro (Fig. 3G). To visualize USP7-KDM4A protein complex in live settings, we carried out immunofluorescence (IF) assay in T24 cells that exposed the intracellular co-localization of USP7 and KDM4A proteins (Fig. 3H). To detail USP7-KDM4A specific protein interaction mechanisms, we pursued molecular docking prediction assay for modeling (Fig. 3I). Based on the model, a panel of expression vectors were generated to express Flag versus HA tagged USP7 and KDM4A constructs, as indicated (Supplementary Fig. S1I). Upon transfection of these constructs into 293 T cells, co-IP tests revealed that the USP7 561-1102aa fragment binds to full-length KDM4A (Fig. 3J), while the KDM4A 886-1064aa fragment binds to full-length USP7 (Fig. 3K).

### USP7 stabilizes KDM4A proteins by catalyzing deubiquitination and uncoupling the proteasome-mediated degradation pathway

**Fig. 4:**
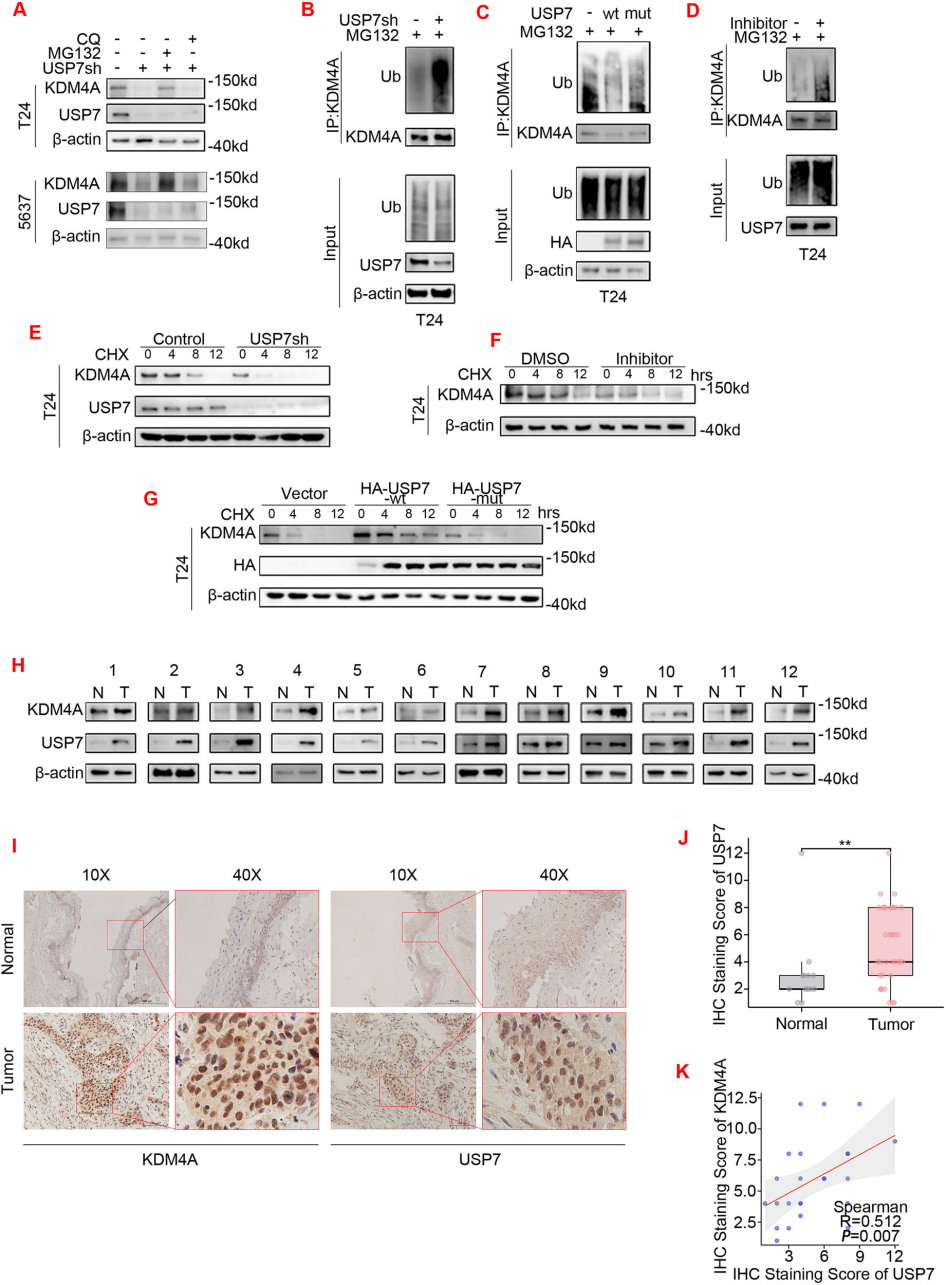
USP7 stabilizes KDM4A proteins via deubiquitination and blockade of the proteasome-mediated degradation pathway. **A** T24 and 5637 cells were infected with lentiviral control versus USP7-specific shRNA (sh#1) expression vectors. Upon 24 h treatment with MG132 (20 μM) versus CQ (20 μM), total proteins were normalized for WB assay. **B** T24 cell were infected with lentiviral control versus USP7-specific shRNA (sh#1) expression vectors. Next, cells were treated with MG132 (20 μM) for 12 h and cell lysates were subjected to IP with KDM4A antibody for WB assay. **C** similarly to (**B**), T24 cell were infected with lentiviral vectors (empty vector control, HA-USP7-WT, versus HA-USP7-mutant). Upon 12 h treatment with MG132 (20 μM), the cell lysates were subjected to IP with the KDM4A antibody for WB assay. **D** T24 cell were pre-treated with DMSO versus USP7 inhibit (P5091, 30uM) for 24 h, and then treated with MG132 (20 μM) for additional 12 h. The cell lysates were subjected to IP with the KDM4A antibody for WB assay. **E** T24 cell were infected with lentiviral control versus USP7-specific shRNA (sh#1) expression vectors. Cells were next treated for indicted time course with cycloheximide (CHX, 5 μM) and total proteins were normalized for WB assay. **F** Similarly to (E), T24 cells were pre-treated with DMSO versus USP7 inhibitor (P5091, 30uM) for 24 h. Cells were next treated for indicted time course with cycloheximide (CHX, 5 μM) and total proteins were normalized for WB assay. **G** T24 cells were transfected with control (empty vector) versus HA-USP7-WT or HA-USP7-mutant expression vector. Cells were next treated for indicted time course with cycloheximide (CHX, 5 μM) and total proteins were normalized for WB assay. **H** 12 pairs of bladder cancer tissues (T) and normal bladder mucosal epithelium (N) were freshly collected. Total proteins were normalized for WB analysis. **I** Representative images of IHC staining for KDM4A and USP7 proteins in 37 cases of bladder cancer versus 10 cases of normal bladder tissues. **J**, **K** Statistics analysis of USP7 IHC staining in 37 cases of bladder cancer versus 10 cases of normal bladder tissues (**J**) and its correlation with KDM4A expression levels in cancer cases (R = 0.512, p = 0.0017, Spearman).

Deubiquitinating enzymes (DUB) are proteases that catalyze the cleavage of protein-ubiquitin bonds to reverse the action of ubiquitin E3 ligases. Accordingly, we reason USP7 acts as a DUB to mitigate KDM4A protein degradation by deubiquitination and disruption of proteasome-specific pathway. To test the hypothesis that USP7 stabilizes KDM4A proteins via its enzymatic activity, we first confirmed that in T24 and 5637 cells USP7-KD did not change KDM4A mRNA levels (Supplementary Fig. S1J), suggesting transcription-independent mechanisms. Next, we examined two canonic protein degradation pathways (autophagy versus proteasome) on KDM4A protein expression, using specific inhibitors (MG132 for proteasome versus chloroquine (CQ) for autophagy). As shown, in both T24 and EJ cell lines USP7-KD decreased KDM4A protein expression, an effect that was effectively reversed by MG132 (but not CQ) treatment (Fig. 4A). To specifically address the deubiquitination mechanism, we then showed that KDM4A ubiquitination modification was indeed increased upon USP7-KD in T24 cells (Fig. 4B). In contrast, the levels of KDM4A ubiquitination was attenuated by over-expression (OE) of USP7 wild-type (WT), but not its catalytic mutant (USP7-C223S) (Fig. 4C). Consistently, an increase in KDM4A ubiquitination also occurred in T24 cells upon treatment with a USP7-specific inhibitor (P5091) (Fig. 4D).

The above observations are concordant on the mechanism that the USP7-KDM4A protein complex functions to sustain KDM4A protein stability via deubiquitination that uncouples the proteasome pathway. To further confirm this mechanism, we next monitored the degradation rate of KDM4A protein in T24 cells treated with cycloheximide (CHX) to block new protein synthesis. We found that in T24 cells, interference with USP7 expression using siRNA or inhibition of USP7 function with P5091 accelerated KDM4A protein degradation, while overexpression of USP7-WT (but not the USP7-C223S mutant) had the opposite effect (Fig. 4E–G). Notably, we next summoned clinical samples to assess the correlation between USP7 and KDM4A protein expression and performed WB assay on normalized proteins from 12 surgically resected bladder cancer tissue cases. As shown, both USP7 and KDM4A proteins were over-expression in the tumors (Fig. 4H). Moreover, a validation test with IHC staining of 37 bladder cancer tissues and 10 normal bladder tissues showed over-expressed KDM4A and USP7 proteins were in positive correlation (Fig. 4I–K).

### USP7 drives bladder cancer cell proliferation through KDM4A protein stabilization

**Fig. 5:**
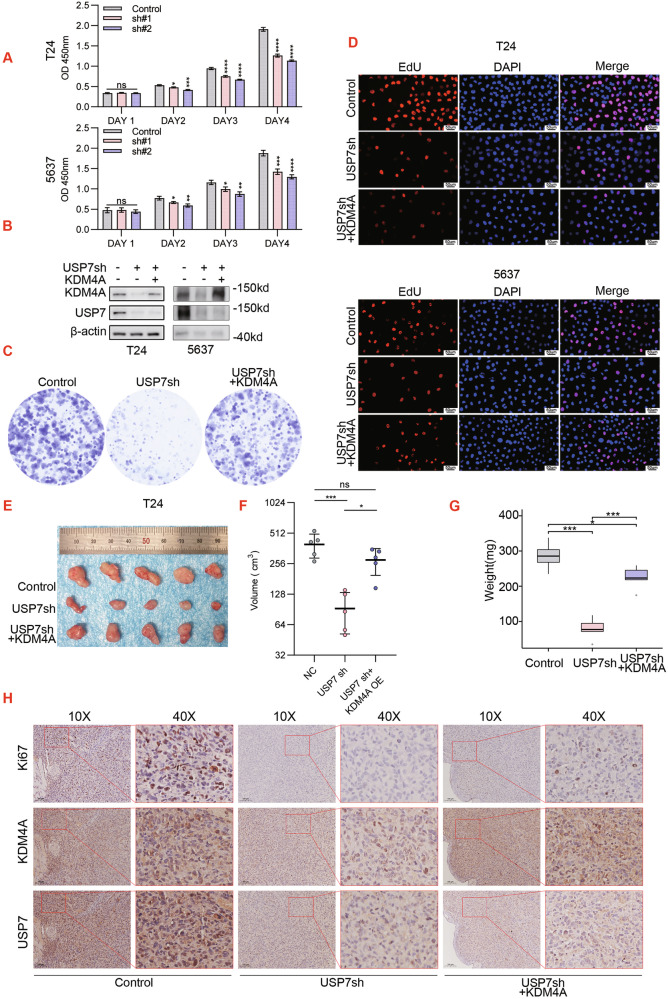
USP7 drives bladder cancer cell proliferation through KDM4A protein stabilization. **A** T24 and 5637 cells were infected with lentiviral control versus USP7-specific shRNA (sh#1 and sh#2) expression vectors. Cell viability was detected by CCK8 assay. **B** T24 and 5637 cells were infected with lentiviral control vector, USP7-specific shRNA (sh#1) expression vector, and KDM4A over-expression (OE) vector as indicated. Total proteins were normalized for WB assay. The above cell lines (**B**) were seeded 2000 cells/well for cell cloning assay (**C**) and were subjected to EdU assay (**D**). The above established T24 stable lines (**B**) were subjected to generate mice xenografts, followed by assessing the in vivo effects on tumor growth (**E**), tumor volume (**F**), tumor weight (**G**), and IHC staining (**H**). (* P < 0.05, **P < 0.01, *** P < 0.001, **** P < 0.0001*, ns=no sign)*.

Our above findings ratified the notion that USP7-KDM4A form a protein complex in which KDM4A proteins are isolated by USP7 from the proteasome-mediated degradation pathway. Next, we designed a panel of biological tests to explore cellular functions of the USP7-KDM4A module in bladder cancer. Accordingly, we infected T24 and 5637 cells with lentiviral USP7 shRNA (sh#1 and sh#2) and showed that USP7-KD mitigated bladder cancer cell proliferation in CCK8 assays (Fig. 5A), plate cloning assays (Supplementary Fig. S2A, B), and EdU assays (Supplementary Fig. S2C, D). In functional compensation assays, we established three test groups with lentiviral infection of T24 and 5637 cell lines: control (empty vector), USP7-KD (sh#1 expression vector), and USP7-KD + KDM4A-OE (KDM4A over-expression vector) (Fig. 5B). As shown, USP7-KD inhibited cell proliferation and this inhibitory effect was partially reversed by KDM4A-OE in CCK8 assay (Supplementary Fig. S2E), plate cloning assays (Fig. 5C and Supplementary Fig. S2G, H), and EdU assays (Fig. 5D and Supplementary Fig. S2F). To validate these findings in the in vivo settings, we generated mouse T24 xenograft and demonstrated that, consistently, USP7-KD elicited inhibition of tumor growth was partially reversed by KDM4A-OE (Fig. 5E–G). Ki67 IHC staining similarly showed that the decline in Ki67 positivity by USP7-KD was partially restored upon KDM4A-OE (Fig. 5H). Taken together, our cellular and animal tests uphold the conclusions that USP7 has pro-proliferative functions in bladder cancer that are partially mediated by KDM4A protein stabilization.

### USP7 interference mitigates cisplatin resistance in bladder cancer cells via KDM4A down-regulation

**Fig. 6:**
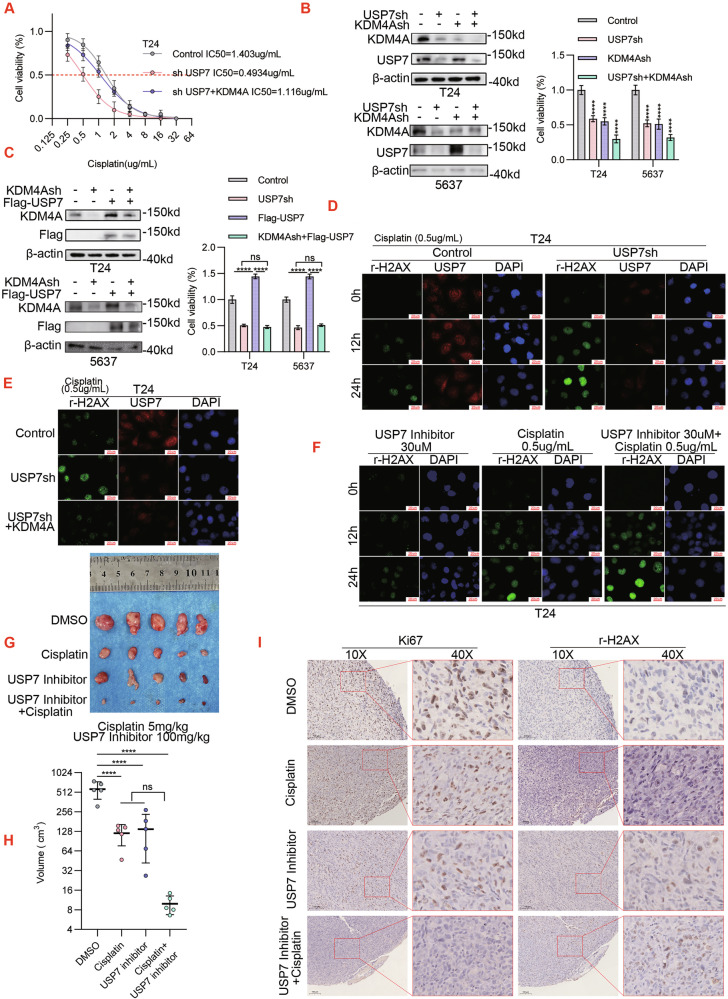
USP7 interference mitigates cisplatin resistance in bladder cancer cells via KDM4A down-regulation. **A** T24 cell were infected with lentiviral control vector, USP7-specific shRNA (sh#1) expression vector, and KDM4A over-expression (OE) vector as indicated. Cells were next challenged by a range of cisplatin doses to measure IC50 values. **B** T24 and 5637 cells were infected with lentiviral control vector, USP7-specific shRNA (sh#1) expression vector, and KDM4A-specific shRNA (sh#1) expression vector as indicated. Total proteins were normalized for WB assay (left panel) while CCK8 assay was performed to detect cell viability upon 48 h of treatment with cisplatin. (Cisplatin 0.5ug/mL) (right panel). **C** T24 and 5637 were transfected with null virus, KDM4A knockdown lentivirus and Flag-USP7, and KDM4A and Flag-USP7 were detected by Western blot after co-transfection with KDM4A knockdown lentivirus and Flag-USP7. CCK8 assay was performed to detect cell viability of T24 and 5637 cells after 48 h of treatment in cisplatin (Cisplatin 0.5ug/mL). **D** T24 cells were infected with lentiviral control vector versus USP7specific shRNA (sh#1) expression vector as indicated. Cells were next treated with cisplatin (0.5ug/mL) for immunofluorescence (IF) assay (100x; green: r-H2AX; red: USP7; blue: nuclei, DAPI). **E** (Supplementary Fig. 3B) T24 and 5637 cells were infected with lentiviral control vector, USP7-specific shRNA (sh#1) expression vector, and KDM4A over-expression (OE) vector as indicated. Cells were next challenged for 24 h with cisplatin (0.5ug/mL) for IF assay. **F** (Supplementary Fig. 3D) T24 and 5637 cells were treated with 30uM of USP7 inhibitor (P5091) and/or 0.5ug/mL of cisplatin for indicated time points for IF assay. **G–I** T24 cells were subjected to mice xenograft generation, followed by treatment for 4 weeks with vehicle (DMSO), cisplatin (5 mg/kg*week), and/or USP7 inhibitor (P5091, 100 mg/kg* week) as indicated, followed by assessment of tumor growth (**G**), tumor volume (*** *p* < 0.001) (H), and IHC staining (10x versus 40x, I). (* P < 0.05, **P < 0.01, *** P < 0.001, **** P < 0.0001, *ns=no sign)*.

Cisplatin is the mainstream chemotherapeutic agent for advanced bladder cancer and here we specifically assessed the effect of targeting USP7-KDM4A axis on cisplatin resistance. First, we monitored IC50 upon 48 h of cisplatin exposure, using T24 and 5637 cell lines that were established with lentiviral infection: control (empty vector), USP7-KD (sh#1), and USP7-KD + KDM4A-OE. As shown, USP7-KD decreased the cisplatin IC50 value, an effect that was partially reversed by KDM4A-OE (Fig. 6A and Supplementary Fig. S3A). Next, we performed USP7 versus KDM4A complementary tests in T24 and 5637 cells to address their relevance to cisplatin sensitivity. As shown, the outcomes of CCK8 assay showed that either USP7-KD or KDM4A-KD alone sensitized cell viability to cisplatin treatment, whereas USP7 and KDM4A double-KD further aggravated cisplatin sensitivity (Fig. 6B). In contrast, USP7-OE enhances the resistance of bladder cancer cells to cisplatin, while USP7-OE combined with KDM4A-KD can offset the cisplatin resistance of bladder cancer cells caused by USP7 overexpression (Fig. 6C).

Cisplatin insult would induce DNA damage, which is marked by phosphorylation of histone H2AX at serine 139 (pH2AX-Ser139) to form γ-H2AX. Next, we tracked the γ-H2AX signal as an indicator of cisplatin sensitivity with immunofluorescence (IF) assay. Indeed, IF tests showed that USP7-KD led to time-dependent increase of γ-H2AX in T24 and 5637 cells (Fig. 6D and Supplementary Fig. S3C–F). In comparison, KDM4A-OE neutralized the increase in γ-H2AX expression upon USP7-KD (Fig. 6E and Supplementary Fig. 3B–G). Similarly, the USP7 inhibitor P5091 also elicited γ-H2AX signals in T24 and 5637 cells along the time-course of treatment, while P5091 and cisplatin co-treatment further intensified γ-H2AX expression (Fig. 6F and Supplementary Fig. 3D–H). To monitor the in vivo effects on cisplatin sensitivity, T24 mouse xenograft was subjected to treatments with P5091 (100 mg/kg) and/or cisplatin (5 mg/kg). As shown, each compound had inhibitory effect on tumor growth, and their combination conferred profound anti-tumor activities (Fig. 6G, H and Supplementary Fig. 3E). In accordance, Ki-67 IHC staining tests on P5091 and cisplatin revealed that in the tumor xenograft, each compound had anti-proliferation activities and their combination further aggravated the extent of growth inhibition (Fig. 6I). Collectively, these in vitro and in vivo tests ratified that targeting the USP7-KDM4A axis would sensitize cisplatin responsiveness in bladder cancer.

## Discussion

Bladder cancer represents one of the most prevalent malignancies worldwide, with treatment efficacy substantially limited by drug resistance, which severely compromises patient outcomes [1, 3, 6] [33]. As a cornerstone chemotherapeutic agent, cisplatin demonstrates modest therapeutic effects in bladder cancer, but its clinical utility is markedly constrained by acquired resistance. Current research has identified multiple cisplatin resistance mechanisms, including enhanced DNA repair capacity, overexpression of drug transporters, and aberrant activation of key signaling pathways [34–36]. Emerging evidence highlights the growing significance of protein post-translational modification enzymes, particularly demethylases and deubiquitinases, in bladder cancer pathogenesis and drug resistance [12]. The lysine demethylase KDM4A, a crucial epigenetic regulator, exhibits aberrant activation in various malignancies [16, 37]. Our study not only confirmed KDM4A overexpression in bladder cancer tissues and its correlation with patient prognosis but also functionally demonstrated its regulatory role in bladder cancer cell proliferation. Notably, KDM4A knockdown significantly suppressed tumor growth in xenograft models, providing a theoretical foundation for developing KDM4A-targeted therapies.

Dysregulation of the ubiquitin-proteasome system, particularly ubiquitination and deubiquitination processes, plays a pivotal role in bladder cancer progression [38–40]. Among deubiquitinases, USP7 has garnered substantial attention for its critical functions in various cancers [13, 41–43]. Through systematic molecular interaction studies, we elucidated the functional relationship between USP7 and KDM4A: mass spectrometry initially identified their potential interaction, which was subsequently confirmed by co-immunoprecipitation and GST pull-down assays. Truncation experiments further pinpointed the critical interaction domains (amino acids 886-1064 of KDM4A and 561-1102 of USP7), providing important insights into their molecular interplay.

We demonstrated that USP7 regulates KDM4A stability via the proteasomal pathway: CHX chase assays showed accelerated KDM4A degradation upon USP7 silencing, while ubiquitination assays confirmed that wild-type (but not mutant) USP7 removes ubiquitin modifications from KDM4A. Clinical sample analysis revealed significant co-expression patterns of USP7 and KDM4A in bladder cancer tissues, suggesting their potential synergistic role in tumor progression.

The malignant progression of bladder cancer closely correlates with tumor cell proliferation, metastasis, and chemotherapy resistance [33]. Functional studies showed that USP7 knockout markedly inhibited bladder cancer cell proliferation and xenograft growth, effects that could be partially reversed by KDM4A overexpression. Importantly, we found that knockdown of either KDM4A or USP7 reduced the IC50 of cisplatin in bladder cancer cells, while KDM4A overexpression enhanced cisplatin resistance. Dual knockout of USP7/KDM4A showed synergistic effects in improving cisplatin sensitivity, and γ-H2AX detection confirmed that USP7 inhibitors potentiate cisplatin-induced DNA damage. Animal studies further validated the therapeutic potential of USP7 inhibitors, demonstrating that combined treatment with USP7 inhibitors and cisplatin significantly suppressed xenograft growth compared to monotherapy.

To date, proteasome inhibitors including bortezomib (Velcade), carfilzomib (Kyprolis), and ixazomib (Ninlaro) have become cornerstone therapies for multiple myeloma and mantle cell lymphoma [44]. Our study demonstrated that the USP7 inhibitor P5091 effectively targets KDM4A downregulation and enhances cisplatin’s antitumor efficacy in bladder cancer. Previous studies have shown P5091’s effectiveness in inhibiting pancreatic, colorectal, and non-small cell lung cancers [45–47], highlighting its promising potential as an anticancer agent.

In summary, this study elucidates the molecular mechanism by which USP7 stabilizes KDM4A through deubiquitination to promote cisplatin resistance in bladder cancer, providing novel targeted strategies to overcome clinical cisplatin resistance. The remarkable synergistic effects of P5091 and cisplatin combination therapy underscore the significant translational potential of these findings.

## Supplementary information

Supplementary Information

Supplementary Figure Legend.

Supplementary figure 1

Supplementary figure 2

Supplementary figure 3

Supplementary Tables 1 and 2

aj-checklist

Raw data for WB

## Data Availability

The original contributions presented in the study are included in the main article and the supplementary material. Further inquiries can be directed to the corresponding authors.
