# Supplementary material for: USP7 promotes chemotherapy resistance and DNA damage response through stabilizing and deubiquitinating KDM4A in bladder cancer
Source: Cell Death Dis. 2025 Dec 23;17(1):123. doi: 10.1038/s41419-025-08297-2 (PMC12847834; doi:10.1038/s41419-025-08297-2)
Supplement: Supplementary file 1 — Supplementary Information [file 41419_2025_8297_MOESM1_ESM.docx]

**Supplementary Materials**

**Supplementary Materials and Methods**

**Antibodies and reagents**

The anti-HA antibody (Cat No. A00241), anti-Ub antibody (Cat No. A00241), anti-β-actin antibody (Cat No. A00241) and anti-ki67 antibody (Cat No. GB111141-100) were purchased from Servicebio Technology Co. (Wuhan, China). The anti-γ-H2AX antibody (trade code A00241) was purchased from PhD Bioengineering Co. (Wuhan, China). All experiments were performed according to the manufacturer's recommendations.

**Plasmids and cloning**

Flag-KDM4A and HA-USP7 expression plasmids were generated by cloning them into the pcDNA3 vector. And the different structural domains of KDM4A and USP7 were cloned into vector pcDNA3-Flag and vector pcDNA3-HA. The RNF41 mutant was generated using the QuikChange II Site-Directed Mutagenesis Kit. Primers used for cloning were as follows:

| KDM4A full length  F:ATGGCTTCTGAGTCTGAAAC  R:CTCCATGATGGCCCGGTATA | KDM4A 1-885aa  F:ATGGCTTCTGAGTCTGAAAC  R:CTTGTGCCGAAAGCAGGTAA |
| --- | --- |
| KDM4A 886-1064aa  F:ATGATTCCTAATTTGGAGCGTG  R:CTCCATGATGGCCCGGTATA | USP7 full length  F:ATGAACCACCAGCAGCA  R:GTTATGGATTTTAATGGCCTTTTCA |
| USP7 55-208aa  F:ATGGCGGAGGAGGACATG  R:CTTTGAATCCCACGCAACT | USP7 209-560aa  F:ATGAAGCACACAGGCTACG  R:TTCCTGCCGCTCCTTC |
| USP7 561-1102  F:ATGGCCCATCTCTATATGCAAG  R:TCCGTTATGGATTTTAATGGCCTTTTCA |  |

**ShRNA lentiviral vector packaging and transduction**

Lentiviral vectors ShRNA-KDM4A, ShRNA-USP7, KDM4A and ShRNA-GFP pLKO1 (control vector) were purchased from QEgene Biotechnology Co. in Shanghai, China. Lentiviral vectors encoding ShRNA were packaged in 293T cells by PEI 40K transfection. Supernatants containing lentiviral particles were collected 48h after transfection. The postviral supernatant was filtered using a sterile 0.45 um filter tip, and T24 cells and EJ cells were transduced with supernatant in the presence of polyglutamine (8 μg/mL) for 24 h and then replaced with fresh medium. Cells were analysed at 48 or 72 h post-transfection.

Lentiviral knock-down sequences:

| KDM4A  shRNA1：GCACCGAGTTTGTCTTGAAAT  shRNA2：GCCTTGGATCTTTCTGTGAAT  shRNA3：GCTGCAGTATTGAGATGCTAA | USP7  shRNA1：CCTGGATTTGTGGTTACGTTA  shRNA2：GTGTCCTATATCCAGTGTAAA  shRNA3：CCAGCTAAGTATCAAAGGAAA |
| --- | --- |

**SiRNA transfections**

SiRNA was mixed with SiRNA using Lipo-2000 and SiRNA was delivered to T24 cells and EJ cells using serum-free medium for 6 h. Cells were analysed at 48 or 72 h post-transfection.

SiRAN sequences：

| HECTD4 | siRNA | GCGTCAGACACATTGACTATT |
| --- | --- | --- |
| HERC2 | siRNA | ACUGUAGCCAGAUUGAAA |
| RANBP2 | siRNA | GCTTGTCAGAATCCAGGTAAA |
| UBA1 | siRNA | GTGCTATGGTTTCTATGGTTA |
| UBA2 | siRNA | GCACCAGATGTCCAAATTGAA |
| USP34 | siRNA | CTTATAGCACATGCGTTTATT |
| USP7 | siRNA | CGTGGTGTCAAGGTGTACTAA |
| SUMO1 | siRNA | GAATGGAGGAAGAAGATGTGA |

**Quantitative real-time PCR (qRT-PCR)**

Primers for qPCR analysis of human gene transcripts were:

| KDM4A |  |
| --- | --- |
| F: | AGGAGAGTGAACTGCCTCCA |
| R: | GGTCTCCTTCCTCTCCATCC |
| β-actin |  |
| F: | TCTCCCAAGTCCACACAGG |
| R: | GGCACGAAGGCTCATCA |
| HECTD4 |  |
| F: | TGTAGTGCTGGAGAGCGACTTG |
| R: | CTGTCTTCGGTCAGCCTGCAAA |
| HERC2 |  |
| F: | AGACACGGTGTCCGACGAGTAT |
| R: | TCTCCGTCACAACAGCACCAGT |
| RANBP2 |  |
| F: | GAAGAGACTGCTCTGGAAGGCT |
| R: | AAGACCGTGAGTCAGTGCCTGA |
| UBA1 |  |
| F: | GCTGGTGCTTGAGCTGTG |
| R: | GTGTGGAGAGGGGTGGAC |
| USP7 |  |
| F: | GTCACGATGACGACCTGTCTGT |
| R: | GTAATCGCTCCACCAACTGCTG |
| USP34 |  |
| F: | CGACTTAGATGCCTTGGCAAGAC |
| R: | GGAGTCCTGTAAGCCCATCATC |
| RANBP2 |  |
| F: | ACAGGTGGGACTGAAGTGATG |
| R: | ATCTTGCTTTCCCCTTGGCT |
| SUMO1 |  |
| F: | CAACTGAGGACTTGGGGGAT |
| R: | ACCCTCAAAGAGAAACCTGAGT |

**Cell viability assay**

T24 and EJ cells were inoculated in 96-well plates with 5000 cells/well, and the assay using CCK8 reagent was carried out after the cells were attached to the wall. CCK8 reagent was mixed with serum-free medium in the ratio of 1:9 and added to the cells and incubated for 60 min, followed by detection of 450 nm absorbance using an enzyme marker.

**cell cloning assay**

T24 and EJ cells were inoculated in 6-well plates at 2000 cells/well, cultured for 7 days, fixed using 4% formalin and stained with crystal violet.

**Edu proliferation assay**

Cell proliferation experiments were performed using the EdU Proliferation Kit from Xavier, 5000 cells/well of T24 cells and EJ cells were inoculated into 96-well plates, and the EdU incubation solution was added for 2 hrs by the half-exchange method as recommended by the reagent company, and then the cell membranes were fixed using 4% formalin, fixed and permeabilised using 0.5% Triton-100, and the nuclei were labelled using DAPI staining. Finally, images of EdU-labelled proliferating cells were captured using fluorescence microscopy, and ImageJ software analysed the percentage of EdU-positive cells for statistical analysis.

Protein immunoprecipitation (IP) and Liquid Chromatography-MS Analysis

Cell lysates were captured on Flag-M2 beads or protein A/G agarose beads (Santa Cruz Biotechnology, Inc. (CA, USA)). The complexes were then separated and the gels were detected by silver staining or Western blotting. For liquid chromatography-mass spectrometry analysis, immunoprecipitation of KDM4A antibody was performed as described above. The precipitated proteins were eluted three times with lysis buffer. Eluted sample solutions were trypsin digested, analysed by liquid chromatography-mass spectrometry, and proteins were identified using the Mascot (v2.3.02) program and compared with the Uniprot Human Protein Database (December 2014 release).

Ubiquitination assay

Cells were transfected with the indicated plasmids and lysed with immunoprecipitation buffer. Immunoprecipitation: 2 mg protein was incubated with the indicated antibodies overnight at 4°C, then protein A/G beads were added and incubated for 2 hours. Beads were washed once with TBS, 1% Triton X-100, 1% SDS, twice with 0.5 M LiCl, TBS buffer, and twice with PBS containing 1% Triton X-100 buffer. Proteins were loaded onto 8% SDS-PAGE gels and immunoblotted with the indicated antibodies.
