# Supplementary material for: USP7 promotes chemotherapy resistance and DNA damage response through stabilizing and deubiquitinating KDM4A in bladder cancer
Source: Cell Death Dis. 2025 Dec 23;17(1):123. doi: 10.1038/s41419-025-08297-2 (PMC12847834; doi:10.1038/s41419-025-08297-2)
Supplement: Supplementary file 2 — Supplementary Figure Legend. [file 41419_2025_8297_MOESM2_ESM.docx]

**Supplementary Fig.S1**

**(A)** Statistical plots of the proliferative capacity of T24 and 5637 cells after knockdown of KDM4A expression by plate cloning assayed. Plate cloning assay in this panel is related to Fig.2C.

**(B)** Statistical plots of the proliferative capacity of T24 and 5637 cells after knockdown of KDM4A expression by EdU assayed.

**(C)** IC50 of 5637 cells for 48 hours in cisplatin.

**(D)** Western blot of over-express KDM4A in T24 and 5637 cells.

**(E-F)** Plate cloning assay of T24 and 5637 cells after over-express of KDM4A.

**(G)** IC50 of 5637 cells for 48 hours in cisplatin after over-express of KDM4A.

**(H)** Eight molecules identified by mass spectrometry as potentially interacting with KDM4A were knocked down using siRNA knockdown followed by RT-qPCR to identify knockdown efficiency (relative to β-actin).

**(I)** Based on the molecular docking results, two KDM4A(K1 and K2) and three USP7 (U1, U2 and U3) truncated fragment patterns were constructed.

**(J)** RT-qPCR was used to detect the changes in the relative β-actin expression levels of USP7 and KDM4A in Lentiviral knockdown of T24 (left) and 5637 (right) cells.

*(* P<0.05, **P<0.01, *** P<0.001, **** P<0.0001, ns=no sign)*

**Supplementary Fig. S2**

**(A)** Plate cloning assay was performed to detect the clone forming ability of T24 and 5637 cells after stable knockdown of USP7 by lentivirus, 2000 cells/well.

**(B)** Statistical graph of clone formation ability of T24 and 5637 cells with knockdown of USP7.

**(C)** EdU assay was performed to detect the proliferative capacity of T24 and 5637 cells after stable knockdown of USP7 by lentivirus.

**(D)** Statistical plots of the proliferative capacity of T24 and 5637 cells after knockdown of USP7 expression by EdU assayed.

**(E)** CCK8 assay to detect the proliferation ability of lentiviral empty vector group, lentiviral knockdown USP7 group and lentiviral knockdown USP7+ over-expression KDM4A group.

**(F)** Statistical plots of proliferative capacity detected by EdU assay in lentiviral empty vector group, lentiviral knockdown USP7 group and lentiviral knockdown USP7+ over-expressing KDM4A group .

**(G)** Cell cloning assay for proliferative capacity of lentiviral empty vector group, lentiviral knockdown USP7 group and lentiviral knockdown USP7+ over-expressing KDM4A group in EJ for 2000 cells/well.

**(H)** Statistical plots of cell clone formation ability in lentiviral empty vector group, lentiviral knockdown USP7 group and lentiviral knockdown USP7+ over-expressing KDM4A group.

*(* P<0.05, **P<0.01, *** P<0.001, **** P<0.0001, ns=no sign)*

**Supplementary Fig. S3**

**(A)** 5637 cell were infected with lentiviral control vector, USP7-specific shRNA (sh#1) expression vector, and KDM4A over-expression (OE) vector as indicated. Cells were next challenged by a range of cisplatin doses to measure IC50 values.

**(B)** Immunofluorescence was examined for r-H2AX changes in control (empty virus), lentiviral knockdown USP7 group and co-transfected USP7 knockdown + KDM4A over-expression group of EJ cell at 24 hrs in 0.5ug/mL cisplatin (100x lens, green: r-H2AX, red: USP7, blue: nuclei, DAPI).

**(C)** Immunofluorescence detection of r-H2AX changes in 5637 cell after lentiviral knockdown of USP7 at 0.5ug/mL cisplatin at 0 hrs, 12 hrs, and 24 hrs (100x magnification, green: r-H2AX, red: USP7, blue: nuclei, DAPI).

**(D)** Immunofluorescence was performed to examine the changes of r-H2AX in EJ cell with 30uM USP7 Inhibitor (P5091), 0.5ug/mL cisplatin, or 0.5ug/L cisplatin + 30uM USP7 Inhibitor (P5091) at 0 h, 12 h, and 24 h. (100x lens, green: r-H2AX. blue: nuclei, DAPI).

**(E)** Statistical chart of subcutaneous tumors collected and weighed at week 6 of tumor inoculation..

**(F-H)** Western blot of r-H2AX expression in T24 and 5637 cells after treat with cisplatin or USP7 Inhibitor (P5091).

*(* P<0.05, **P<0.01, *** P<0.001, **** P<0.0001, ns=no sign)*

**Supplementary table 1**

Clinical baseline data table for KDM4A expression in TCGA-BLCA samples

**Supplementary table 2**

Clinical baseline data table for IHC staining scores of pathological tissue specimens
