# Supplementary figures and images for: USP7 promotes chemotherapy resistance and DNA damage response through stabilizing and deubiquitinating KDM4A in bladder cancer
Source: Cell Death Dis. 2025 Dec 23;17(1):123. doi: 10.1038/s41419-025-08297-2 (PMC12847834; doi:10.1038/s41419-025-08297-2)

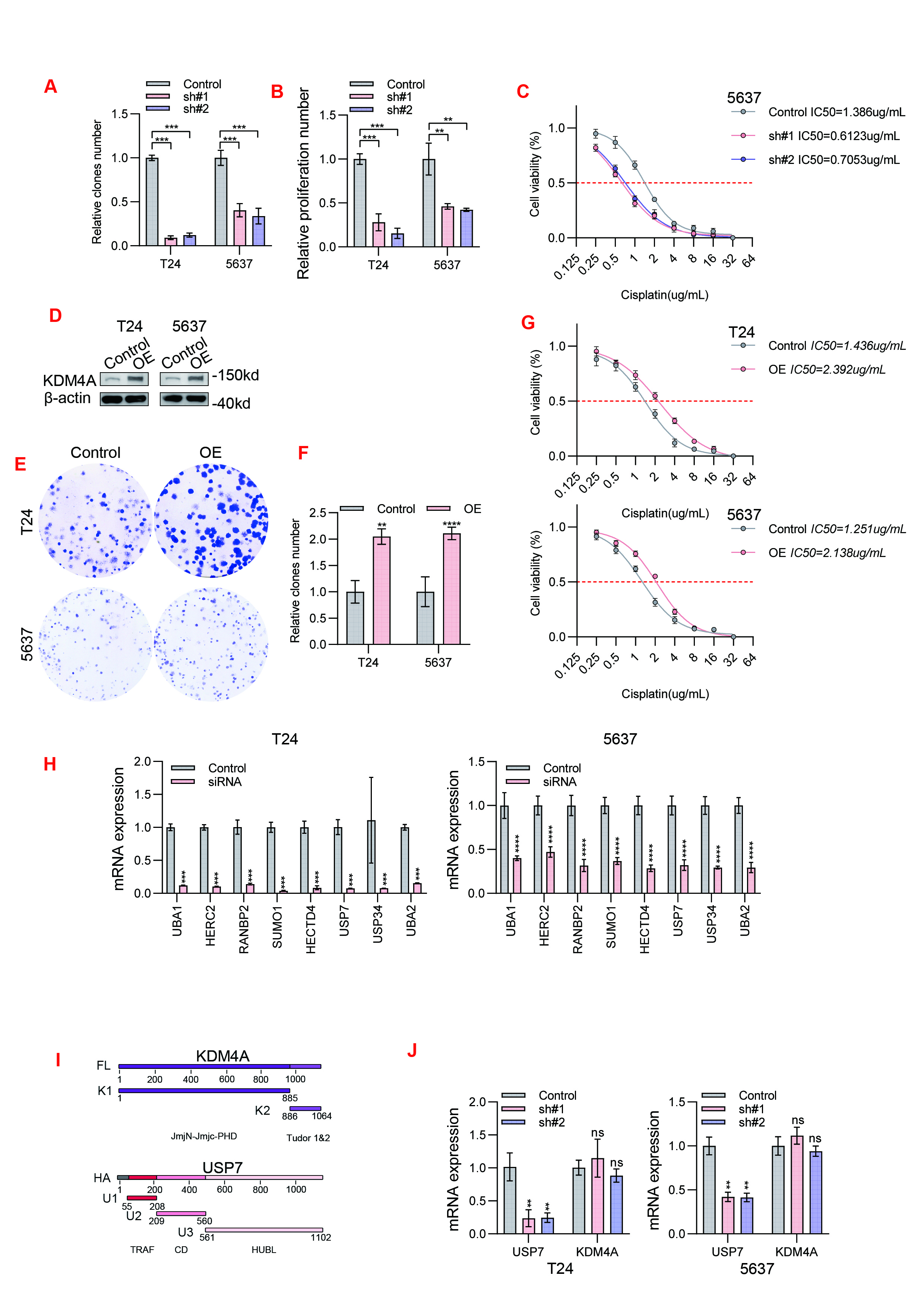

Supplement: Supplementary file 3 — Supplementary figure 1 [file 41419_2025_8297_MOESM3_ESM.jpg]

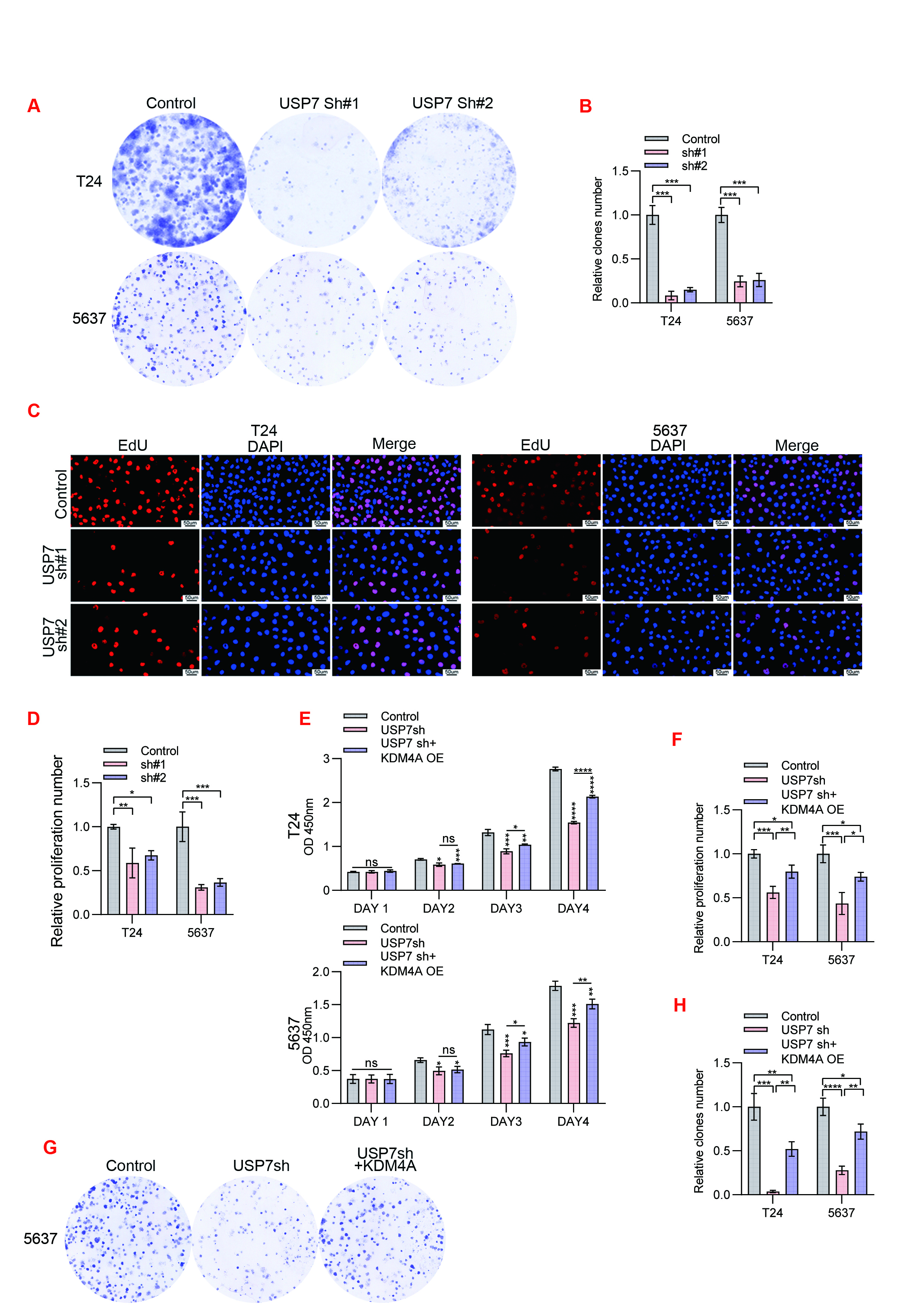

Supplement: Supplementary file 4 — Supplementary figure 2 [file 41419_2025_8297_MOESM4_ESM.jpg]

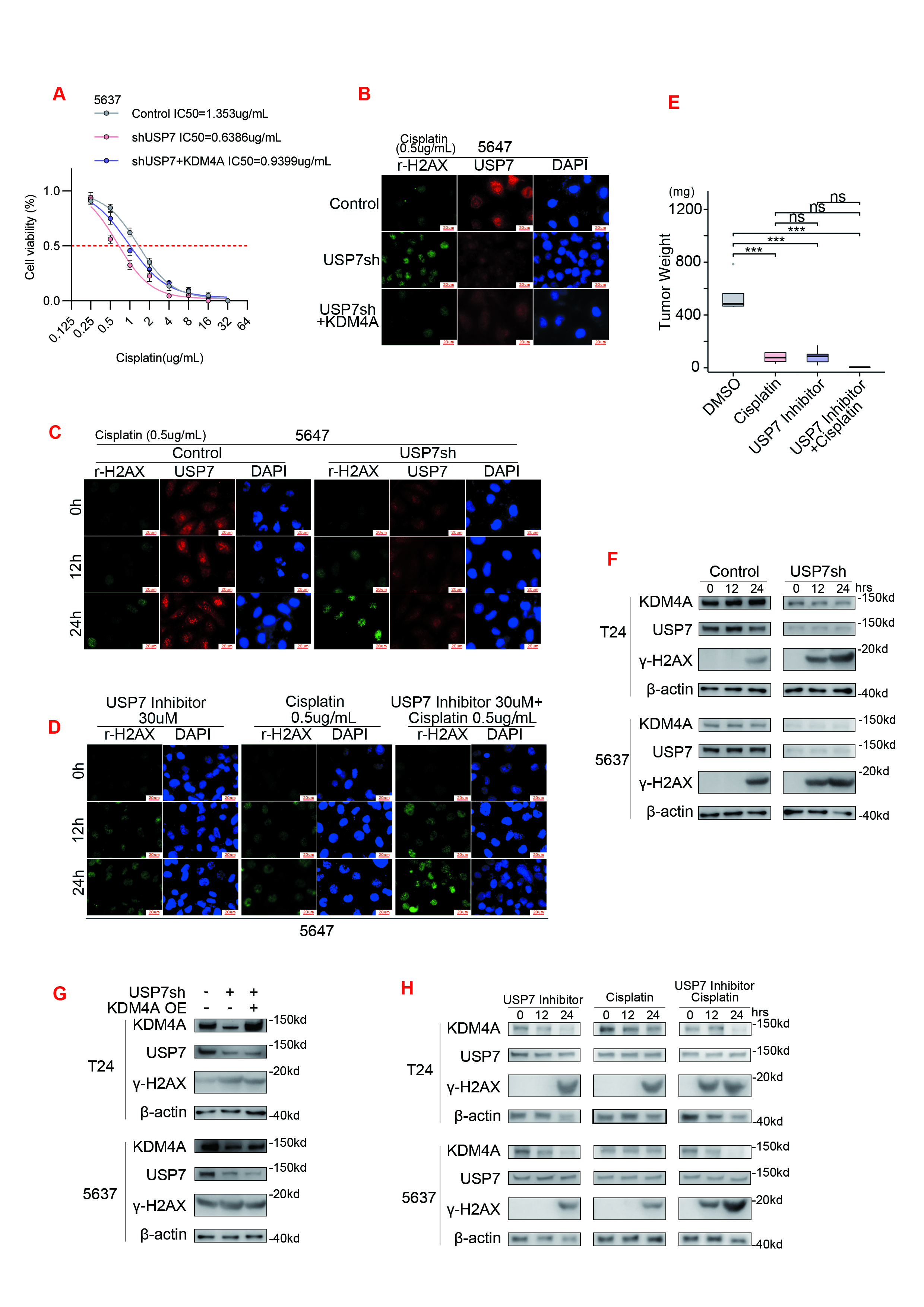

Supplement: Supplementary file 5 — Supplementary figure 3 [file 41419_2025_8297_MOESM5_ESM.jpg]
