# Supplementary material for: USP7 promotes chemotherapy resistance and DNA damage response through stabilizing and deubiquitinating KDM4A in bladder cancer
Source: Cell Death Dis. 2025 Dec 23;17(1):123. doi: 10.1038/s41419-025-08297-2 (PMC12847834; doi:10.1038/s41419-025-08297-2)
Supplement: Supplementary file 6 — Supplementary Tables 1 and 2 [file 41419_2025_8297_MOESM6_ESM.docx]

**Supplementary table 1 & 2**

**Supplementary table 1**

**Clinical baseline data table for KDM4A expression in TCGA-BLCA samples**

| Characteristics | Low expression of KDM4A | High expression of KDM4A | P value |
| --- | --- | --- | --- |
| n | 206 | 206 |  |
| Pathologic T stage, n (%) |  |  | 0.231 |
| T1&T2 | 58 (15.3%) | 65 (17.2%) |  |
| T3&T4 | 137 (36.2%) | 118 (31.2%) |  |
| Pathologic N stage, n (%) |  |  | 0.441 |
| N0 | 120 (32.6%) | 118 (32.1%) |  |
| N1&N3&N2 | 71 (19.3%) | 59 (16%) |  |
| Pathologic M stage, n (%) |  |  | 0.312 |
| M0 | 87 (41%) | 114 (53.8%) |  |
| M1 | 7 (3.3%) | 4 (1.9%) |  |
| Histologic grade, n (%) |  |  | 0.109 |
| Low grade | 7 (1.7%) | 14 (3.4%) |  |
| High grade | 199 (48.7%) | 189 (46.2%) |  |
| Gender, n (%) |  |  | 0.179 |
| Female | 60 (14.6%) | 48 (11.7%) |  |
| Male | 146 (35.4%) | 158 (38.3%) |  |
| Age, n (%) |  |  | 0.427 |
| <= 70 | 112 (27.2%) | 120 (29.1%) |  |
| > 70 | 94 (22.8%) | 86 (20.9%) |  |

**Supplementary table 2**

**Clinical baseline data table for IHC staining scores of pathological tissue specimens**

| Characteristics | Low expression of KDM4A | High expression of KDM4A | P value |
| --- | --- | --- | --- |
| n | 48 | 23 |  |
| T.stage, n (%) |  |  | 0.755 |
| 1&2 | 29 (40.8%) | 13 (18.3%) |  |
| 3&4 | 19 (26.8%) | 10 (14.1%) |  |
| N.stage, n (%) |  |  | 0.697 |
| 0 | 29 (40.8%) | 15 (21.1%) |  |
| 1&2 | 19 (26.8%) | 8 (11.3%) |  |
| M.stage, n (%) |  |  | 0.787 |
| 0 | 34 (47.9%) | 17 (23.9%) |  |
| 1 | 14 (19.7%) | 6 (8.5%) |  |
| Histologic.stage, n (%) |  |  | 0.205 |
| Low stage | 9 (12.7%) | 1 (1.4%) |  |
| High stage | 39 (54.9%) | 22 (31%) |  |
| Age, mean ± sd | 64.229 ± 9.3154 | 63.913 ± 6.8418 | 0.885 |
| Gender, n (%) |  |  | 0.472 |
| Male | 27 (38%) | 15 (21.1%) |  |
| Female | 21 (29.6%) | 8 (11.3%) |  |
