# Supplementary material for: USP7 promotes chemotherapy resistance and DNA damage response through stabilizing and deubiquitinating KDM4A in bladder cancer
Source: Cell Death Dis. 2025 Dec 23;17(1):123. doi: 10.1038/s41419-025-08297-2 (PMC12847834; doi:10.1038/s41419-025-08297-2)

Figure 1 G

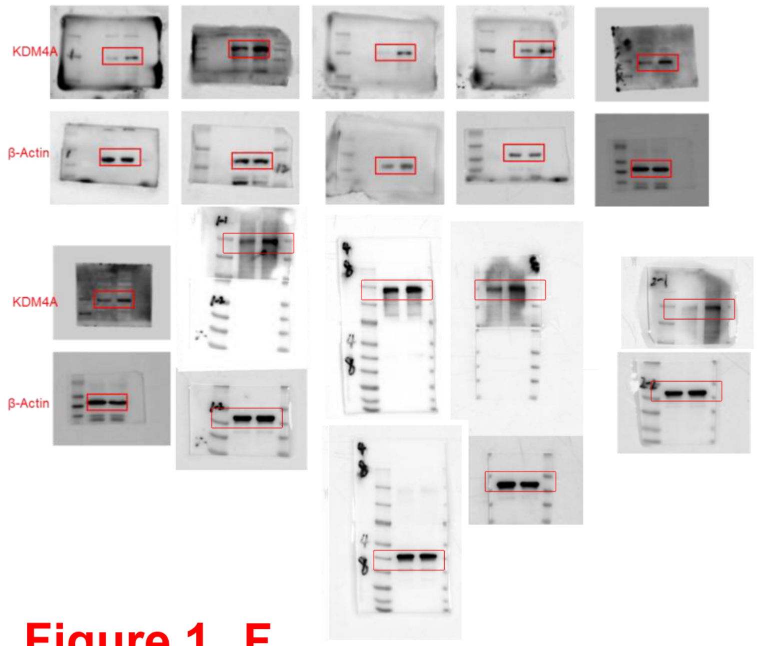

Figure 2 A

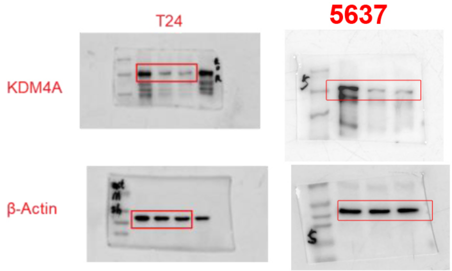

Figure 3C

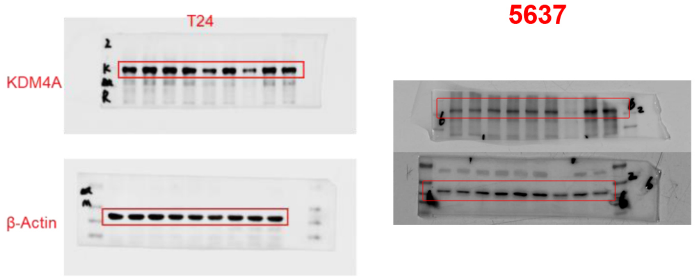

Figure 1 F

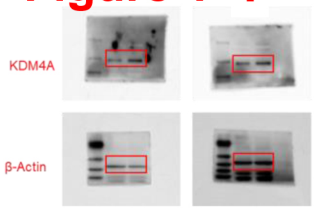

Figure 3F

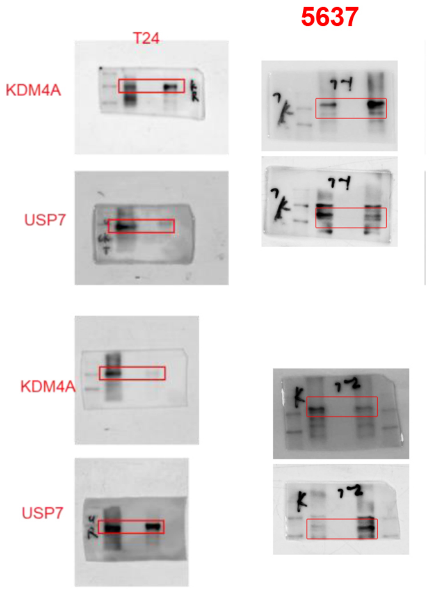

Figure 3E

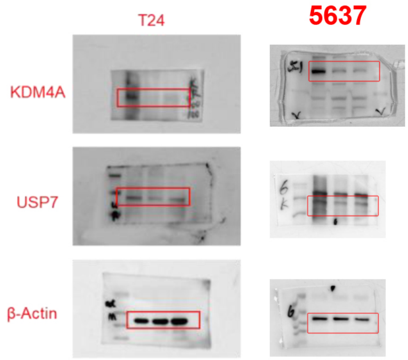

Figure 3G

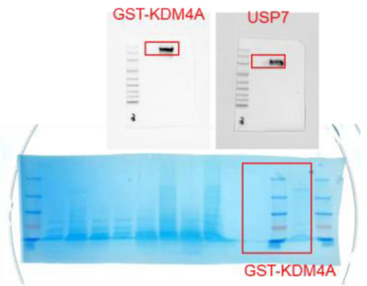

Figure 3J

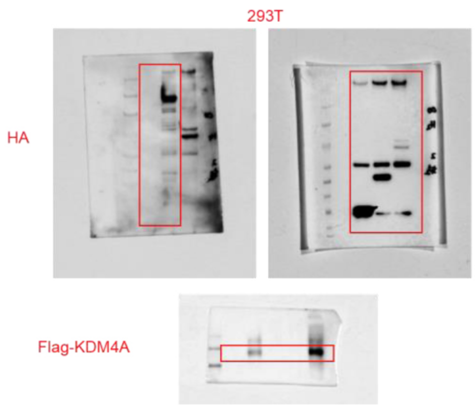

Figure 3K

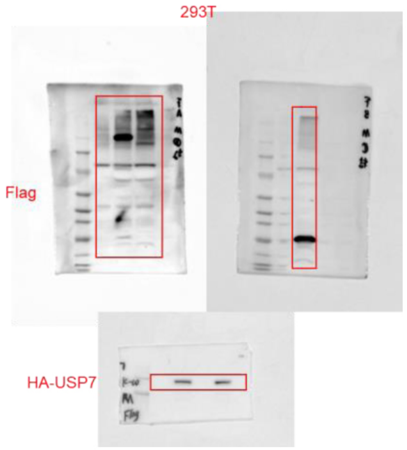

Figure 4A

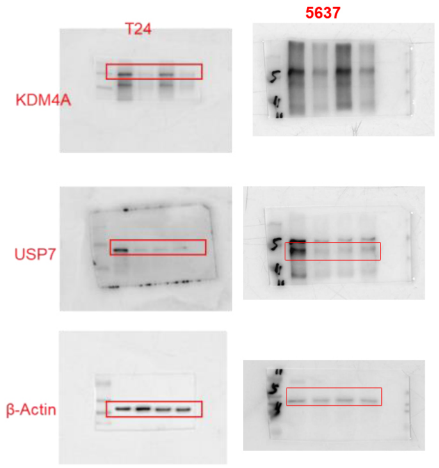

Figure 4D

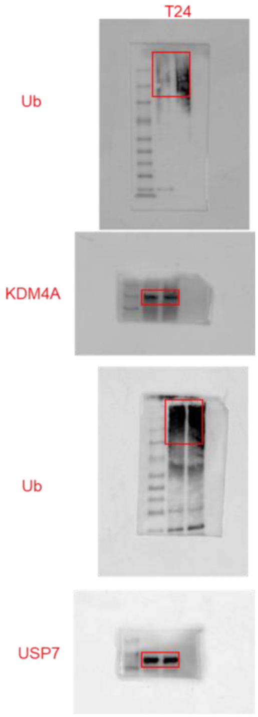

Figure 4B

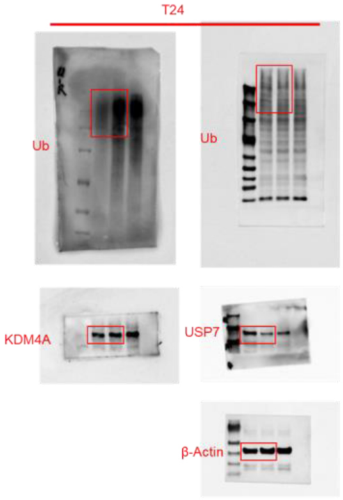

Figure 4E

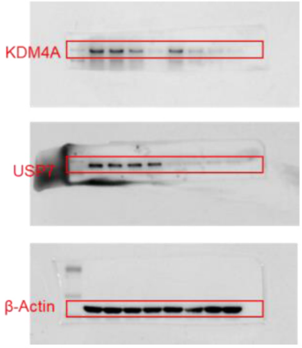

Figure 4C

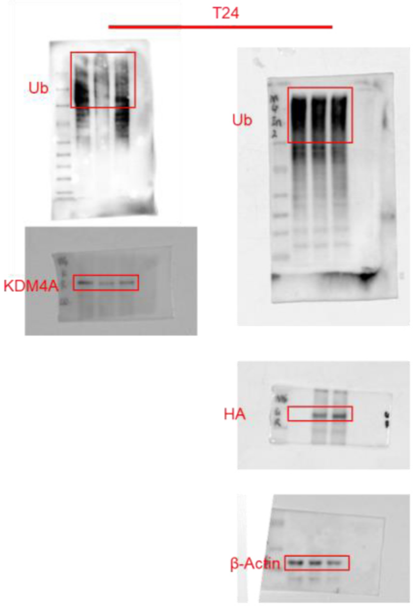

Figure 4F

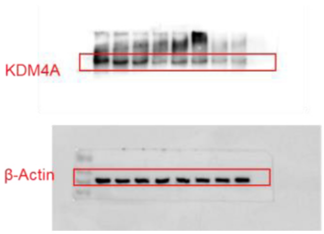

Figure 4G

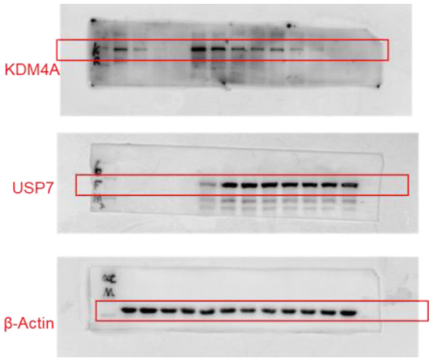

Figure 5B

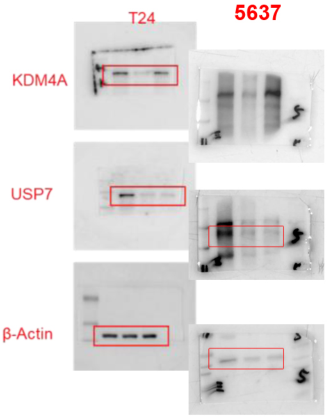

Figure 6C

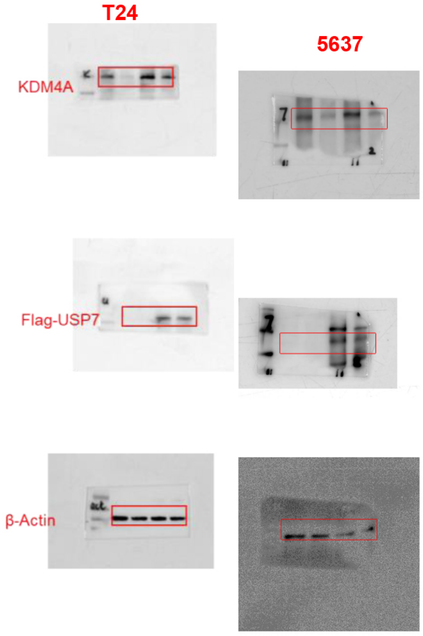

Figure 4H

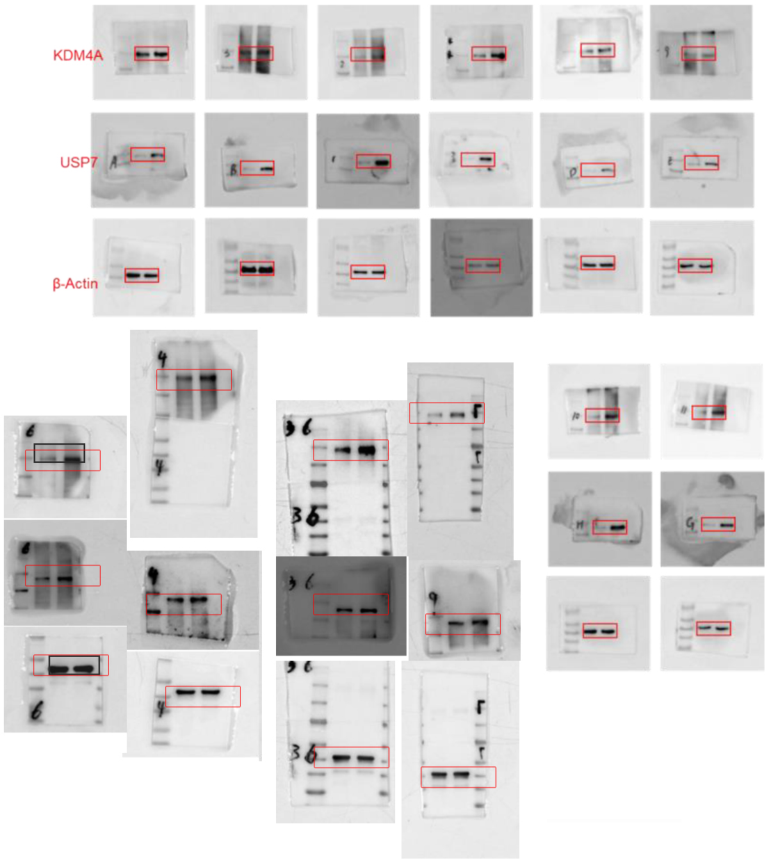

Figure 6B

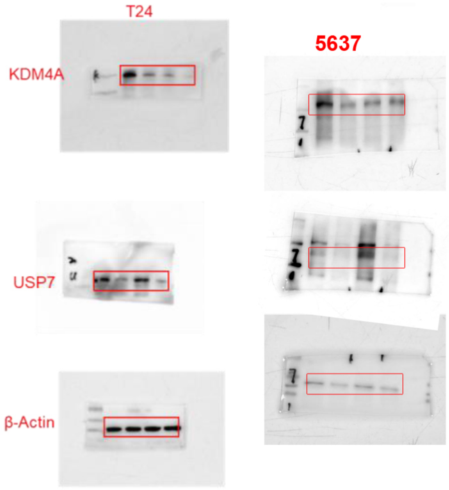

Supplementary Fig 1D

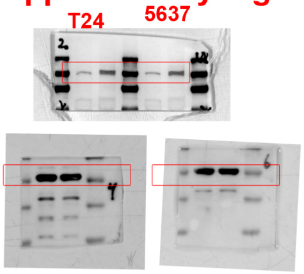

Supplementary Fig 3F

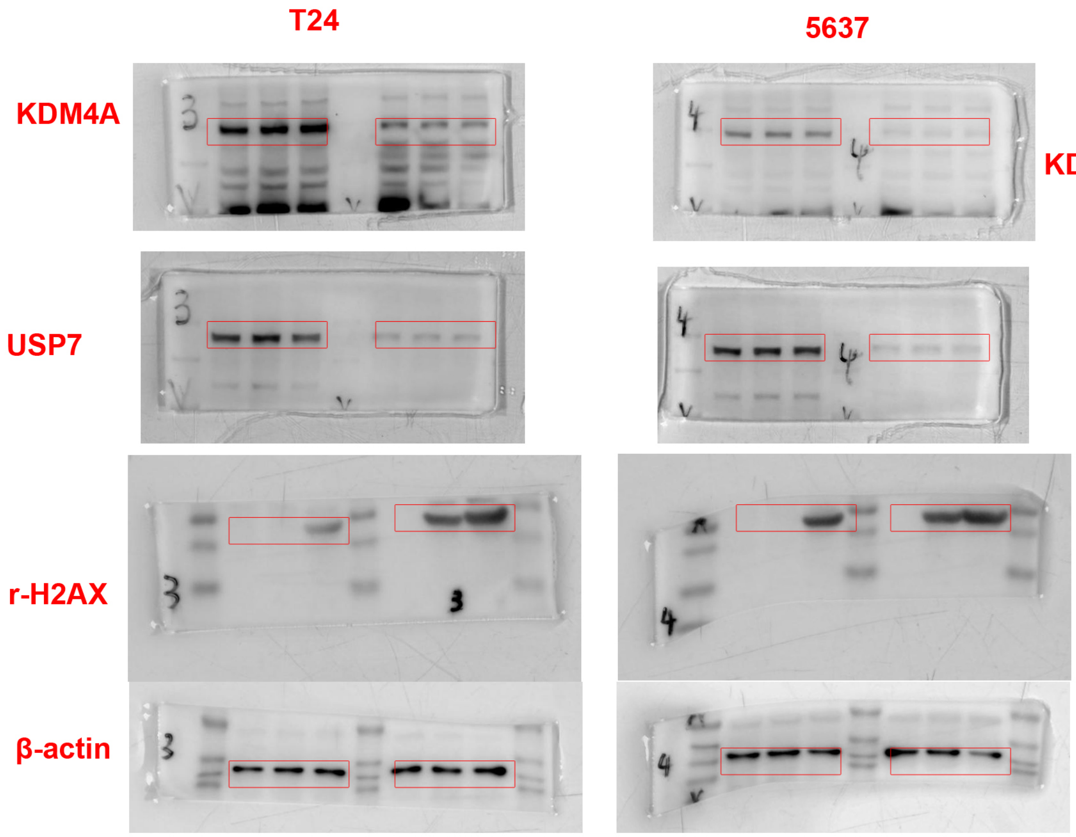

Supplementary Fig 3G

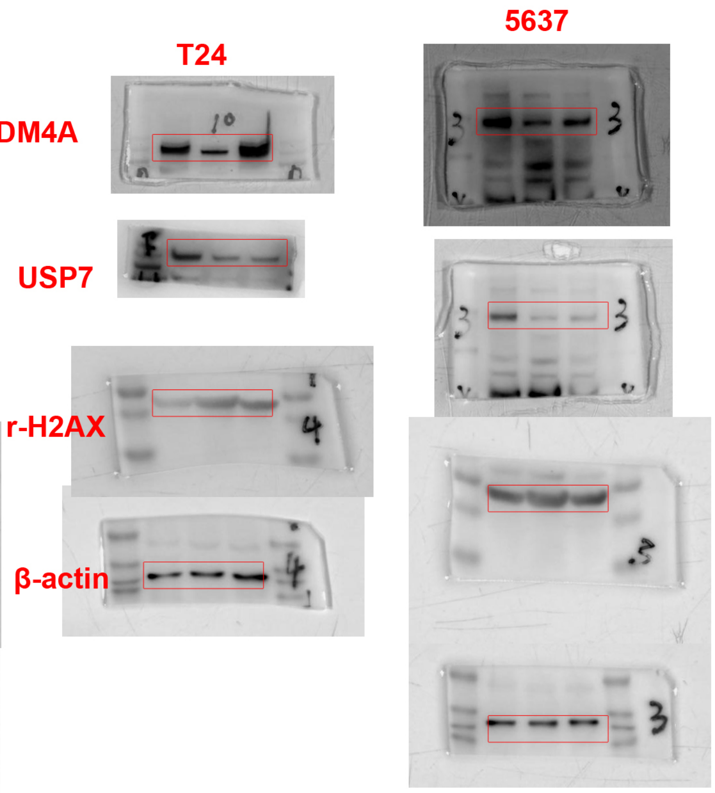

Supplementary Fig 3H

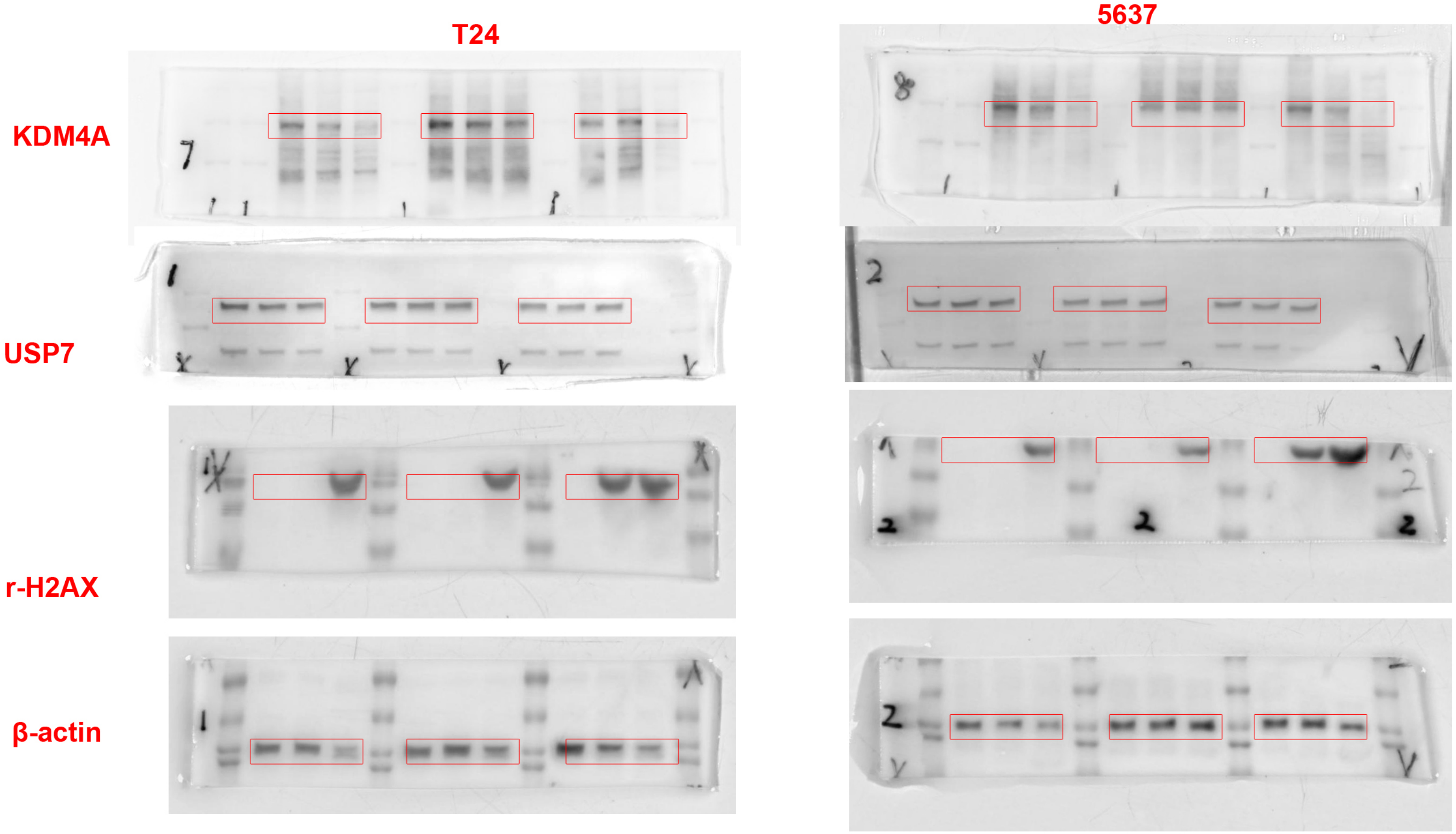

Supplement: Supplementary file 8 — Raw data for WB [file 41419_2025_8297_MOESM8_ESM.pdf]
